# Risk of Venous Thromboembolism with Pemafibrate in Dyslipidemia: A Nationwide, Retrospective, Cohort Study Using a Japanese Claims Database

**DOI:** 10.1007/s43441-025-00883-y

**Published:** 2025-10-23

**Authors:** Kenichiro Ikeda, Mika Tada, Shun Nakano, Takuma Tsushio, Yoshinari Watanabe, Sara Minamikawa, Chieko Ishiguro, Kenji Yokoyama, Kenji Fujisawa, Masaya Tanahashi, Hideki Suganami, Atsushi Kasano

**Affiliations:** 1 Medical Affairs Department, Kowa Company Ltd., Tokyo, Japan; 2 Pharmacovigilance Department, Kowa Company Ltd., Tokyo, Japan; 3 Clinical Data Science Department, Kowa Company Ltd., Tokyo, Japan; 4 Laboratory of Clinical Epidemiology, Department of Data Science, Center for Clinical Sciences, Japan Institute for Health Security, Tokyo, Japan; 5 Department of Hematology and Oncology, Tokai University Hachioji Hospital, Tokyo, Japan; 6 Present Address: Business Coordination Department, Kowa Pharmaceuticals America, Inc., AL, USA

**Keywords:** Venous thromboembolism, Pemafibrate, Selective peroxisome proliferator-activated receptor α modulator, Claims database, Dyslipidemia, Japan

## Abstract

**Aims:**

The study aimed to assess whether pemafibrate, a selective peroxisome proliferator-activated receptor α modulator, increases the risk of venous thromboembolism (VTE) in real-world clinical practice in Japan.

**Methods:**

In this retrospective cohort study, we utilized a claims database with data from December 2017 to July 2023. The exposed group consisted of patients with dyslipidemia using pemafibrate, while the control group included patients not using fibrate drugs. Each exposed patient was randomly matched with five control patients at a 1:5 ratio using time-matching. The primary endpoint was the number of days until the first occurrence of VTE, which was defined using ICD-10 codes and anticoagulant prescription records. We used a Cox proportional hazards model with standardized mortality ratio weight (SMRW) to estimate the adjusted hazard ratio (HR) and 95% confidence interval (CI) for the exposed group relative to the control group.

**Results:**

The study included 23,195 patients in the exposed group and 115,975 in the control group. In the full analysis population, 46.6% of patients were women, with a median age of 70.0 years and a median BMI of 23.6. VTE occurred in 1.2% (286/23,195) of the exposed group and 2.0% (2297/115,975) of the control group, with an incidence rate of 0.95 and 1.33 per 100 person-years, respectively. There was no significant increase in the risk of VTE in the exposed group (HR, 0.925; 95%CI, 0.809–1.058).

**Conclusions:**

We found no increase in VTE risk associated with pemafibrate in clinical practice in Japan.

**Supplementary Information:**

The online version contains supplementary material available at 10.1007/s43441-025-00883-y.

## Introduction

Venous thromboembolism (VTE) is widely recognized as a potentially fatal vascular disease [1]. In the United States, an estimated 1,220,000 cases of VTE occur annually [2]. Of these, pulmonary embolism (PE) is known to be particularly high-risk, with reports in Denmark indicating that up to 20% of acute PE events result in death within 90 days [3]. In Japan, VTE was previously considered uncommon [4]. However, with advancement of diagnostic capabilities and increasing awareness among medical staff, the frequency of VTE diagnosis in clinical practice has been rising [5]. As a result, VTE has now become a major public health concern in Japan just, as it is in Europe and the United States.

Fibrates are used to treat dyslipidemia, activate peroxisome proliferator-activated receptor (PPAR) α, and lower blood triglyceride (TG) levels by promoting lipoprotein metabolism and the uptake of fatty acids in the liver and inhibiting TG synthesis [6]. However, despite these therapeutic benefits, several studies have reported that fibrate treatment can increase the risk of VTE. For example, findings from the Fenofibrate Intervention and Event Lowering in Diabetes (FIELD) study, a large-scale clinical trial, showed a higher incidence of VTE in the fenofibrate group compared with the placebo group [7]. These findings were subsequently supported by a meta-analysis suggesting that fibrate medications increase the risk of VTE [8]. Additionally, the study using VigiBase, the World Health Organization's collaborative global pharmaco-vigilance database, indicated a significant increase in the risk of VTE among hospitalized patients with dyslipidemia who had low VTE risk at baseline and were treated with fenofibrate [9]. However, the mechanism by which fibrates medications increase the risk of VTE remains unclear.

Pemafibrate is a selective PPARα modulator that has demonstrated higher selectivity for PPARα than existing PPARα agonists and that provides a potent TG-lowering effect. Compared with fenofibrate or placebo, pemafibrate enhances TG lowering without increasing adverse events or adverse drug reactions [10, 11]. In the Pemafibrate to Reduce Cardiovascular Outcomes by Reducing Triglycerides in Patients with Diabetes (PROMINENT) trial, which assessed whether treatment with pemafibrate prevents the occurrence of cardiovascular composite outcomes, the safety analysis indicated a significantly higher hazard ratio (HR) for VTE in the pemafibrate group compared with the placebo group [12]. However, an earlier clinical trial of pemafibrate in Japan showed one PE event, without establishing a causal relationship to the drug, and a Japanese post-marketing surveillance study found no occurrences of VTE [13, 14]. These findings highlight inconsistencies regarding the impact of pemafibrate on VTE risk. In addition, clinical studies often focus on specific populations with limited size and demographic characteristics. This emphasizes the need for evaluating VTE risk by using medical databases that reflect real-world clinical practice.

To investigate whether pemafibrate medication increases the risk of VTE in real-world clinical practice in Japan, we used a Japanese claims database to assess the risk of VTE in dyslipidemia patients treated with pemafibrate, in comparison to patients who did not receive any treatment with fibrates.

## Materials and Methods

### Study Design and Database

This retrospective cohort study utilized data from MDV (Medical Data Vision Co., Ltd, Tokyo, Japan), which includes records from Diagnosis Procedure Combination (DPC)-eligible hospitals. The DPC/Per Diem Payment System is a patient classification system developed in Japan for acute inpatient care, aimed at data collection [15]. The database is anonymized and contains claims data and laboratory data covering 45.3 million patients from 493 hospitals as of October 2023. The MDV database is widely utilized by researchers to generate new scientific findings [16, 17]. In this study, we analyzed data on patients for whom medical records between December 1, 2017 and July 31, 2023 were available and who were diagnosed with dyslipidemia at least once.

We configured two groups of patients: a pemafibrate-treated group (the exposed group) and a group not receiving any fibrate medications, including pemafibrate (the control group). We also established a follow-up period to evaluate outcomes in both groups. Fibrate medications are listed in Supplementary Table 1, with the codes for patient inclusion. We designated the start date for the follow-up period as the index date, corresponding to the first date of pemafibrate prescription for the exposed group and to a time-matched date for the control group as described later (Supplementary Fig. 1). For both groups, the 180 days prior to the index date were defined as the baseline period for aggregating background information, to ensure no use of fibrates and no VTE events, and to extract covariate data for analysis.

This study used the intention-to-treat (ITT) dataset for primary analysis and the per-protocol (PP) dataset for secondary analysis. The ITT analysis did not consider treatment changes or duration of exposure when determining effect, whereas both treatment changes and duration of exposure were considered in the PP analysis of effect. For ITT analysis, the follow-up period for both groups ended on either the date of outcome occurrence or the final day of the observable period, which is the last day on which the patient's medical care was recorded. In the PP analysis, we introduced a “gap period” or “grace period” during which the drug’s effect was assumed to continue even if the medication was not being used. The “gap period” was defined as a period between prescriptions, during which the prescription was considered to be ongoing, and the “grace period” was defined as the period after the prescription had ended during which drug exposure might persist. Therefore, in PP analysis for the exposed group, the end of the follow-up period was determined by one of the following four time points: the date the outcome occurred, the last day of the observable period, the date a fibrate other than pemafibrate was prescribed, or the date when pemafibrate exposure ended. For the control group, this was one of three time points: the date of outcome occurrence, the last day of the observable period, or the date on which pemafibrate or another fibrate was prescribed. For both groups, the observable period was from the earliest recorded date of medical care to the last recorded date.

### Participants

This study consisted of patients who had their first recorded diagnosis of dyslipidemia (ICD-10: E78x) on or after June 1, 2018, when pemafibrate became available for prescription in Japan. The codes used for patient inclusion are shown in Supplementary Table 1.

The exposed group was defined as patients with a record of pemafibrate prescription who did not meet any of the exclusion criteria (Table 1). Patients in the control group were selected by time-matching to the exposed group as followed: First, for a specific pemafibrate patient (Patient A), the number of days was calculated from the first diagnosis of dyslipidemia to the date of pemafibrate prescription (X days). For all other patients except Patient A, if it was confirmed that the patient had no prescription records for pemafibrate during the period from X days after the initial dyslipidemia diagnosis and going back 180 days, that patient became a candidate for the control group. The index date was set at X days after each candidate’s first dyslipidemia diagnosis. Patients who met the exclusion criteria were excluded from consideration. Five patients were then selected at random from the remaining patient pool and were matched with Patient A. This process was repeated for each patient in the exposed group. In this study, it was possible under the study protocol for the same patient to be assigned to both the exposed and control groups in both the intention-to-treat (ITT) and per-protocol (PP) analyses. In the ITT analysis, group assignment was based solely on the patient’s status at the start of follow-up, regardless of any changes in prescription status during the follow-up period. In other words, follow-up continued even if pemafibrate was prescribed during the follow-up period, and the time was not partitioned between periods classified as pertaining to the exposed group and the control group. For the PP analysis, just as for the ITT analysis, group assignment was determined by the patient’s status at the start of follow-up. However, changes in prescription status were incorporated so that follow-up time was partitioned: follow-up in the control group was censored at the start of pemafibrate treatment, and follow-up in the exposed group began at that point. As a result, exposure status and follow-up windows were mutually exclusive, preserving the independence of observations in each analytic group for the PP analysis.

**Table 1:** Inclusion and exclusion criteria

Inclusion criteria
1. Having records of first dyslipidemia (ICD-10 codes: E78) diagnosis after June 1, 2018
Exclusion criteria
1. No medical records more than 180 days prior to the index date 2. Having records of any fibrate prescription (ATC code: C10A2) during the baseline period 3. Having records of VTE (ICD-10 codes: I26x, I80x, and/or I82x) diagnosis during the baseline period 4. In the exposed group, having records of pemafibrate prescription before first dyslipidemia (ICD-10 code: E78x) diagnosis after June 1, 2018 5. In the control group, the end of the observable period is before the index date 6. Age is under 20 at the index date 7. Having records of biliary atresia (ICD-10 code: K831) or gallstone (ICD-10 code: K80x) diagnosis during the baseline period 8. Having records of cyclosporine (ATC code: L04X0) or rifampicin (ATC code: J04A1) prescription during the baseline period

ATC, anatomical therapeutic chemical; ICD-10, International Statistical Classification of Diseases and Related Health Problems 10th Revision; VTE, venous thromboembolism

### Covariates

The following covariates were collected during the baseline period and used to estimate the propensity score. The covariates of age, sex, body mass index (BMI), smoking status, hypertension, diabetes mellitus, chronic kidney disease, liver disease, cancer, myocardial infarction, heart failure, atrial fibrillation or atrial flutter, cerebrovascular disease, pneumonia, chronic pulmonary disease, urinary tract infection, connective tissue disease, human immunodeficiency virus (HIV), Crohn's disease or ulcerative colitis, superficial vein thrombosis, varicose vein, coagulopathy, fracture, trauma, injury, surgery excluding transfusion and installation or removal of inferior vena cava (IVC) filter, blood transfusion, central venous line, intravenous catheter or lead, statin, ezetimibe, icosapent ethyl (IPE), ω-3 fatty acid, insulin, metformin, sulfonylurea, α-glucosidase inhibitor, sodium glucose cotransporter 2 (SGLT2) inhibitor, dipeptidyl peptidase-4 (DPP4) inhibitor, glucagon-like peptide-1 (GLP-1) receptor agonist, angiotensin converting enzyme (ACE) inhibitor, angiotensin II receptor blocker (ARB), β blocker, calcium channel blocker, chemotherapy, hormone replacement therapy, platelet aggregation inhibitor, thrombolytic, oral anticoagulant, and parenteral anticoagulation were selected based on the 2019 ESC Guidelines [18] and Japanese guidelines for diagnosis, treatment and prevention of pulmonary thromboembolism and deep vein thrombosis (JCS 2017) [19]. The codes used for covariate selection are provided in Supplementary Table 2.

### Endpoint

The primary endpoint was the number of days until the first onset of VTE during the follow-up period. For outpatients, the outcome was defined as the record of VTE (ICD-10: I26x, I80x, I82x) accompanied by a prescription for a thrombolytic and/or anticoagulant for the same patient at the same facility on the same date. For inpatients, the outcome was defined as having a record of VTE and a thrombolytic and/or anticoagulant prescription recorded during the same period of hospitalization. The outcome occurrence date was defined as the date of the thrombolytic and/or anticoagulant prescription. If there were multiple prescription dates, we used the earliest date after the record of VTE. The codes related to the outcome are listed in Supplementary Table 3.

### Statistical Methods

In this study, we addressed immortal time bias in the exposed group by using time-matching to configure the control group. For patient characteristics in both the exposed and control groups, and for exposure in terms of the number of days pemafibrate was prescribed and the total and average daily prescribed doses in the exposed group, summary statistics were calculated for continuous variables, and frequencies and proportions were calculated for categorical variables. We also calculated the standardized difference to assess covariate balance. We used the standardized mortality ratio weight (SMRW) to adjust for differences in baseline characteristics and improve comparability between the two groups. Propensity scores were calculated after addressing missing covariate data using multiple imputation (MI). SMRW was applied to subsequent calculations using the calculated propensity score. We assessed the distribution of propensity scores before and after using SMRW, and calculated standardized differences (StdDiff) for all covariates to evaluate patient characteristics.

We applied MI to variables with missing data (BMI and smoking), under the missing at random assumption. Binary variables were imputed using logistic regression, and ordinal categorical variables were imputed using ordinal logistic regression, both based on fully conditional specification. Variables for patient characteristics, presence or absence of outcome occurrence, and follow-up period were included in the imputation model. None of the patients had superficial venous thrombosis, so that variable was excluded from the imputation model. After performing 20 imputations, the results from each imputed dataset were combined into a single set of estimates using Rubin’s rules. If the histogram of propensity scores by patient characteristics and the Kaplan–Meier plot of outcome occurrence were consistent across all 20 results, the analysis from the first imputed dataset was used in this paper.

The incidence rate for the outcome was calculated by dividing the number of events by the total person-years of follow-up and was expressed as per 100 person-years. The primary analysis estimated the adjusted HR and 95% confidence interval (CI) for the exposed group relative to the control group, using the ITT dataset and a Cox proportional hazards model with SMRW. The secondary analysis followed the same approach as the primary analysis but used the PP dataset, in which four possible durations (0, 30, 90, or 180 days) were set for the period of residual drug effect (the gap or grace period). The duration of the prescription was defined as the period from the prescription date to the prescription date plus number of prescription days in the relevant prescription record minus 1 day, assuming that drug-taking was initiated on the day of prescription for a single prescription record. The continuation of the prescription was defined as the period from the end date of the dose period for the Xth prescription record to the X plus one prescription record, if the period was shorter than the gap or grace period for multiple prescription records. The subgroup analyses were performed on the primary endpoint. The analyzed subgroups were defined based on the covariates in Table 2.

**Table 2:** Main patient characteristics

		Total N = 139,170	Exposed group N = 23,195	Control group N = 115,975	StdDiff	After MI and SMRW StdDiff: ITT (min, max)
Demographics						
Age, years	Median (IQR)	70.0 (58.0, 78.0)	61.0 (51.0, 71.0)	71.0 (60.0, 79.0)		
	Mean ± SD	67.5 ± 14.3	60.6 ± 13.9	68.9 ± 14.0		
Age category	< Median	67,552 (48.5)	16,158 (69.7)	51,394 (44.3)		
	Median ≤	71,618 (51.5)	7037 (30.3)	64,581 (55.7)		
Age category	20 ≤ , < 30	1391 (1.0)	383 (1.7)	1008 (0.9)	0.070	0.008 (0.008, 0.008)
	30 ≤ , < 40	4255 (3.1)	1302 (5.6)	2953 (2.5)	0.156	0.005 (0.005, 0.006)
	40 ≤ , < 50	11,762 (8.5)	3484 (15.0)	8278 (7.1)	0.253	0.000 (0.000, 0.001)
	50 ≤ , < 60	20,916 (15.0)	5467 (23.6)	15,449 (13.3)	0.267	− 0.006 (− 0.006, − 0.005)
	60 ≤ , < 70	29,228 (21.0)	5522 (23.8)	23,706 (20.4)	0.081	0.001 (0.001, 0.002)
	70 ≤ , < 80	42,199 (30.3)	5212 (22.5)	36,987 (31.9)	− 0.213	− 0.002 (− 0.003, − 0.002)
	80 ≤ , < 90	25,119 (18.0)	1705 (7.4)	23,414 (20.2)	− 0.379	0.002 (0.002, 0.002)
	90 ≤ , < 100	4232 (3.0)	118 (0.5)	4114 (3.5)	− 0.217	0.001 (0.001, 0.001)
	100 ≤	68 (0.0)	2 (0.0)	66 (0.1)	− 0.027	0.000 (0.000, 0.000)
Sex	Female	64,808 (46.6)	7755 (33.4)	57,053 (49.2)	0.324	− 0.006 (− 0.006, − 0.006)
BMI, kg/m^2 a^	Median (IQR)	23.6 (21.1, 26.3)	25.0 (22.7, 27.9)	23.3 (20.8, 25.9)		
	Mean ± SD	24.0 ± 4.3	25.6 ± 4.4	23.6 ± 4.2		
BMI category^a^	< 25	15,155 (64.6)	2163 (50.2)	12,992 (67.8)		
	25 ≤	8314 (35.4)	2148 (49.8)	6166 (32.2)		
BMI category^a^	< 18.5	1870 (8.0)	122 (2.8)	1748 (9.1)	− 0.268	0.004 (0.001, 0.006)
	18.5 ≤ , < 25	13,285 (56.6)	2041 (47.3)	11,244 (58.7)	− 0.229	0.008 (0.005, 0.012)
	25 ≤ , < 30	6376 (27.2)	1521 (35.3)	4855 (25.3)	0.218	− 0.005 (− 0.010, − 0.001)
	30 ≤ , < 35	1529 (6.5)	501 (11.6)	1028 (5.4)	0.226	− 0.005 (− 0.010, 0.000)
	35 ≤ , < 40	318 (1.4)	100 (2.3)	218 (1.1)	0.091	− 0.002 (− 0.008, 0.004)
	40 ≤	91 (0.4)	26 (0.6)	65 (0.3)	0.039	− 0.003 (− 0.009, 0.001)
Smoking history^b^		8665 (39.6)	2048 (51.8)	6617 (36.9)	− 0.304	0.004 (0.001, 0.009)
Medical history						
Hypertension		19,397 (13.9)	3899 (16.8)	15,498 (13.4)	− 0.096	0.044 (0.043, 0.044)
Diabetes mellitus		35,434 (25.5)	8096 (34.9)	27,338 (23.6)	− 0.251	0.038 (0.037, 0.038)
Liver disease		11,516 (8.3)	3086 (13.3)	8430 (7.3)	− 0.200	0.023 (0.023, 0.024)
Cancer		27,349 (19.7)	5008 (21.6)	22,341 (19.3)	− 0.058	0.014 (0.013, 0.014)
Heart failure		17,403 (12.5)	2986 (12.9)	14,417 (12.4)	− 0.013	0.019 (0.019, 0.020)
Pneumonia		21,341 (15.3)	4163 (17.9)	17,178 (14.8)	− 0.085	0.033 (0.033, 0.033)
Concomitant use						
Statin		34,731 (25.0)	5831 (25.1)	28,900 (24.9)	− 0.005	0.030 (0.030, 0.031)
Ezetimibe		4629 (3.3)	1487 (6.4)	3142 (2.7)	− 0.178	0.027 (0.026, 0.027)
IPE		2349 (1.7)	759 (3.3)	1590 (1.4)	− 0.127	0.013 (0.012, 0.013)
ω-3 fatty acid		1217 (0.9)	543 (2.3)	674 (0.6)	− 0.147	0.014 (0.013, 0.015)
Thrombolytic		108 (0.1)	12 (0.1)	96 (0.1)	0.012	0.000 (0.000, 0.000)
Oral anticoagulant		7488 (5.4)	1102 (4.8)	6386 (5.5)	0.034	0.008 (0.008, 0.009)
Parenteral anticoagulant		7971 (5.7)	1315 (5.7)	6656 (5.7)	0.003	0.020 (0.019, 0.020)

Data are given as n (%)

^a^Total N = 23,469, Exposed group N = 4311, Control group N = 19,158

^b^Total N = 21,879, Exposed group N = 3952, Control group N = 17,927

BMI, body mass index; IPE, icosapent ethyl; IQR, interquartile range; ITT, intention-to-treat; MI, multiple imputation; SD, standard deviation; SMRW, standardized mortality ratio weight; StdDiff, standardized differences

This study did not specify a sample size based on power.

All statistical hypotheses were tested at a significance level of 0.05. Two-sided 95% CI was estimated unless otherwise specified. Statistical analyses were performed using SAS version 9.4 or higher (SAS Life Science Analytics Framework version 5.3 or higher).

## Results

### Patient Disposition

**Fig. 1:**
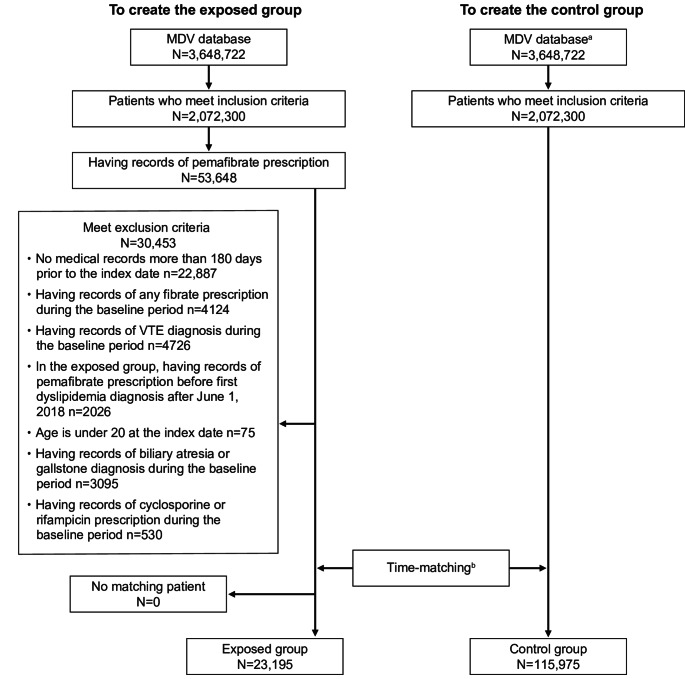
Patient disposition. ^a^The same database was used to create both the exposed group and the control group. ^b^Each exposed patient was randomly matched with five control patients at a 1:5 ratio using time-matching. MDV, medical data vision; VTE, venous thromboembolism

We extracted data for 2,072,300 dyslipidemia patients with medical records available from December 1, 2017 to July 31, 2023. These patients had at least one record of a diagnosis of dyslipidemia on or after June 1, 2018. Among these patients, 53,648 had a record of the prescription of pemafibrate. After applying the exclusion criteria, 23,195 remained in the exposed group. The control group was selected from the initial pool of 2,072,300 patients who met the inclusion criteria, and consisted of 115,975 patients who were matched at a ratio of 5:1 with the exposed group for analysis (Fig. 1).

### Patient Characteristics

The full analysis population consisted of 46.6% women, with a median age of 70.0 years and a median BMI of 23.6 kg/m^2^. Major comorbidities included diabetes mellitus (25.5%), cancer (19.7%), and hypertension (13.9%). A history of smoking was present in 39.6%. Concomitant medications included statin (25.0%), ACE inhibitor/ARB (19.5%), and platelet aggregation inhibitor (14.0%). After imputing missing data and applying SMRW, the value of the absolute StdDiff for covariates in patient characteristics was less than 0.1, indicating well-balanced covariates between the two groups (Table 2, Supplementary Table 4). The distribution of propensity scores mostly overlapped between the exposed and control groups across all 20 imputed datasets (Supplementary Fig. 2).

For the exposed group, the mean duration of the pemafibrate prescription was 373.4 ± 376.4 days, and the mean daily prescribed dose was 0.2 ± 0.1 mg/day. The mean follow-up period was 1.30 years for the exposed group and 1.49 years for the control group in ITT analysis (Supplementary Table 5).

### Endpoints

**Fig. 2:**
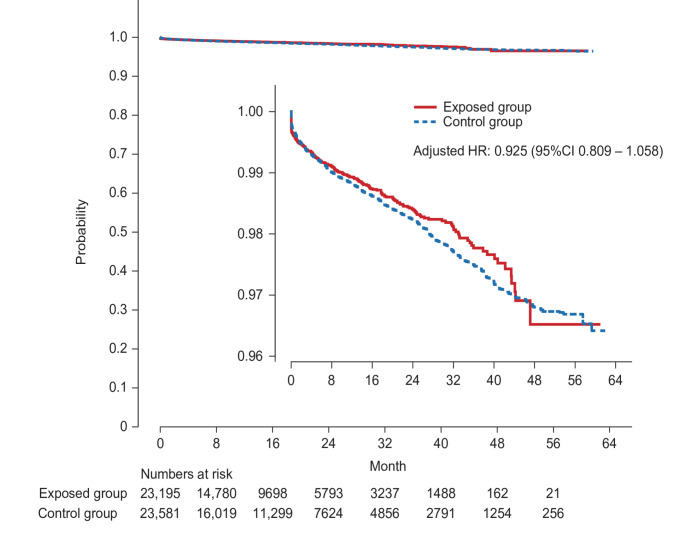
Kaplan–Meier plot of time to first VTE event: ITT analysis. Cox proportional hazards model weighted with SMRW. CI, confidence interval; HR, hazard ratio; ITT, intention-to-treat; SMRW, standardized mortality ratio weight; VTE, venous thromboembolism

**Fig. 3:**
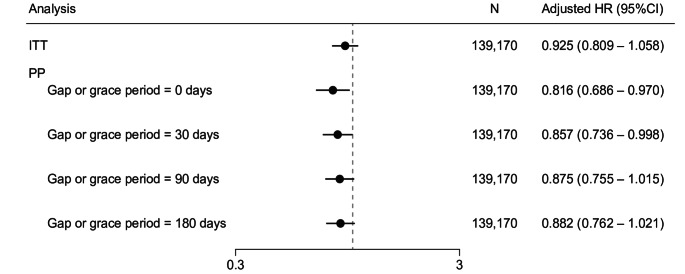
Forest plot of time to first VTE event: ITT analysis and PP analysis. Gap or grace periods for PP analysis are 0, 30, 90, and 180 days. Cox proportional hazards model weighted with SMRW. CI, confidence interval; HR, hazard ratio; ITT, intention-to-treat; PP, per-protocol; SMRW, standardized mortality ratio weight; VTE, venous thromboembolism

In the primary analysis (ITT), VTE occurred in 1.2% (286/23,195) of the exposed group and 2.0% (2297/115,975) of the control group. The incidence rate for VTE was 0.95 per 100 person-years in the exposed group and 1.33 per 100 person-years in the control group (Supplementary Table 5). There was no significant difference in the risk of VTE between the two groups (ITT analysis: adjusted HR 0.925, 95% CI 0.809–1.058) (Fig. 2). In the secondary analysis, we assessed the risk of VTE across four scenarios with varying gap or grace periods. No significant differences were observed in two of these scenarios. The risk reduction of VTE was identified in the scenarios with a 0-day and 30-day gap or grace period; however, this finding lacks clinical significance (Fig. 3 and Supplementary Fig. 3). Kaplan–Meier plots displayed consistent trends across all datasets with the multiple imputation of missing values.

The results of the ITT subgroup analysis were generally consistent with the primary findings except for a few minor subgroups (Supplementary Fig. 4).

## Discussion

The present study utilized a Japanese claims database, including DPC data, health insurance claims, laboratory tests, and other medical records, to investigate the risk of VTE in dyslipidemia patients treated with pemafibrate. Although the study population was derived from DPC-eligible hospitals, which provide acute care, demographic characteristics, such as age, sex, BMI, and concomitant statin use, were similar to those observed in a Japanese post-marketing surveillance of pemafibrate, which provided comprehensive overviews of drug use across a wider range of patient populations [14]. In addition, the average daily dose of pemafibrate in our study closely aligned with the standard daily dose of 0.2 mg in Japan. These findings suggest that the study population was representative of the general population of pemafibrate users in Japan. Furthermore, the absence of a significant difference in VTE incidence rates between the pemafibrate and non-fibrate groups implies that pemafibrate treatment does not increase the risk of VTE in real-world clinical practice in Japan.

To further examine the impact of pemafibrate on the incidence of VTE, we conducted subgroup analyses based on patient characteristics. These analyses showed no clinically meaningful difference in VTE incidence rates between the pemafibrate-treated group and the group not receiving any fibrate medications, including pemafibrate, across all subgroups. This suggests a consistent effect of pemafibrate on the incidence of VTE, regardless of specific comorbidities or concomitant medications (Supplementary Fig. 3).

In the PROMINENT trial, the incidence of VTE was significantly higher in the pemafibrate group than the placebo group [12]. Differences in the patient populations, particularly patient characteristics related to VTE risk, may have contributed to the discrepancy between the PROMINENT results and those from the present study, potentially influencing the effect of pemafibrate medication.

The first factor is the impact of patient characteristics. The PROMINENT trial targeted patients with type 2 diabetes and low HDL-C levels, whereas the inclusion criteria for the present study allowed the enrollment of patients without these conditions. VTE shares several risk factors with cardiovascular diseases, including diabetes, obesity, hypertension, smoking, and elevated plasma homocysteine levels [20, 21]. Notably, the risk of thrombosis increases in the presence of diabetes, which is associated with endothelial cell dysfunction, platelet activation, oxidative stress, and heightened coagulation activity [22]. The proportion of patients with diabetes differed considerably between this study (25.5%) and the PROMINENT trial (100%). Additionally, in individuals with obesity, increased circulating procoagulant microparticles and platelet hyperactivity associated with insulin resistance have been linked to elevated thrombotic potential. Obesity is recognized as a risk factor for VTE [23–25], and obesity prevalence is known to differ between Western and Japanese populations [26]. In the present study, although approximately 83% of BMI data was missing and imputed, the median BMI was 23.6 kg/m^2^ compared with 32 kg/m^2^ in the PROMINENT trial. Similarly, the prevalence of comorbid hypertension differed markedly between the two studies: 13.9% in the present study compared with approximately 91% in the PROMINENT trial. These factors may have contributed to differences in VTE risk at baseline between the two studies. Regarding concomitant lipid-lowering medications, nearly all patients in the PROMINENT trial were receiving statins, with approximately 70% taking high-intensity statins and fewer than 1% receiving IPE therapy. In contrast, in the present study, 25.0% of patients were taking statins, 1.7% were taking IPE, and 0.9% were taking ω-3 fatty acids. These differences in lipid management strategies may have also contributed to variations in VTE risk between the two study populations.

The second factor is the influence of race and/or ethnicity. The PROMINENT trial was a large international multi-center study in which approximately 85% of participants were Caucasian, whereas the present study was conducted using medical records from Japanese hospitals, so the majority of participants were Japanese. A subgroup analysis of the FIELD study identified Caucasian ethnicity as a risk factor for VTE [27], and this finding was supported by a report that Factor V Leiden mutation, which is associated with an increased risk of VTE, is more frequently observed in Caucasian populations [28]. Notably, the Factor V Leiden mutation has not been found in the Japanese population [29–31]. In fact, the Japanese cohort of the PROMINENT trial showed a PE incidence of 0.6% (1/160) in the pemafibrate group and 0% (0/145) in the placebo group, and a deep vein thrombosis incidence of 0.6% (1/160) in the pemafibrate group and 0.7% (1/145) in the placebo group, with no substantial difference in incidence proportion between the two groups [32]. No observed increase in VTE risk was associated with pemafibrate medication in the PROMINENT Japanese subgroup, similar to our study.

Epidemiological studies have suggested that the incidence of VTE is higher in Western populations than in Japanese [4]. However, in the present study, the control group did not receive any fibrate medications, including pemafibrate, and the incidence of VTE was higher in that group (1.33 per 100 person-years) than in the placebo group of the PROMINENT trial (0.21 per 100 person-years). One possible explanation for this discrepancy is the difference in prevalence of comorbid cancer between the study populations at baseline. In the PROMINENT trial, patients with active malignancies within the past 2 years (except for non-melanoma skin cancer and cervical cancer) were excluded from the study. In contrast, 19.7% of patients in the present study had some form of cancer. Cancer is a well-established and potent risk factor for VTE [4] and is included as a component in VTE risk scores [33]. In particular, cancer comorbidity is believed to increase the risk of VTE [34, 35]. The difference between the two study populations in the proportion of patients with cancer may have contributed to the observed differences in VTE incidence rates.

In this study, in particular, analyses were conducted with various gap or grace periods. This approach was adopted because the mechanism by which fibrates affect VTE is unknown, and it is unclear how long the potential effects of the drug would persist after exposure has ended. The ITT analysis assumed that the drug’s effects persisted indefinitely, whereas in the PP analysis, the duration of pemafibrate’s residual effects was examined over four different periods (0, 30, 90, and 180 days). We found that no significant increase in VTE incidence was associated with pemafibrate under any analysis method (Figs. 2, 3, Supplementary Fig. 3). The robustness of these results is supported by the consistency of findings under different assumptions about the duration of the residual effect.

### Limitations

This study has limitations. First, because the database used in this study consists of data from hospitals implementing the DPC system, the findings cannot be generalized to all patients. In particular, DPC-eligible hospitals are often key regional medical institutions, so they tend to treat more severe cases. The patient characteristics described in this study may thus differ from typical patients who receive regular treatment at outpatient clinics. Second, 80% of values for BMI and smoking status were missing in the database, requiring imputation for analysis. The imputation was conducted under the missing at random assumption, as it was unlikely that the missing data for BMI and smoking status were missing completely at random given the nature of the database, and analyses under the missing completely at random assumption could have introduced bias. Following imputation, these values were used as covariates to characterize the study population. Although both BMI and smoking status are critical risk factors for VTE, the substantial proportion of missing data prevented a meaningful discussion of their role in VTE risk. Third, the outcomes in this study were defined by combining diagnostic and treatment records. However, the absence of a validation study could lead to potential overestimation of VTE risk. Fourth, the mean follow-up period was less than 1.5 years, which was shorter than for previously reported studies [7, 12] and may have been insufficient for a comprehensive assessment of VTE risk. A likely reason for the short follow-up period was patient transfers that prevented further tracking. Fifth, since this study was conducted using a claims database, the possibility of unmeasured or unknown confounding factors cannot be ruled out.

## Conclusion

In this study using a Japanese claims database including insurance claims data, no increase in VTE risk was associated with pemafibrate medication in routine clinical practice. However, ongoing evaluation of VTE risk remains essential.

## Supplementary Information

Below is the link to the electronic supplementary material.

Supplementary Material 1

## Data Availability

Data sharing, including sharing of the protocol, is not applicable in this study.
