# Supplementary material for: Risk of Venous Thromboembolism with Pemafibrate in Dyslipidemia: A Nationwide, Retrospective, Cohort Study Using a Japanese Claims Database
Source: Ther Innov Regul Sci. 2025 Oct 23;60(1):274–84. doi: 10.1007/s43441-025-00883-y (PMC12753562; doi:10.1007/s43441-025-00883-y)
Supplement: Supplementary file 1 — Supplementary Material 1 [file 43441_2025_883_MOESM1_ESM.pdf]

Supplementary file

Risk of Venous Thromboembolism with Pemafibrate in Dyslipidemia: A Nationwide, Retrospective, Cohort Study Using a Japanese Claims Database

Kenichiro Ikeda, Mika Tada, Shun Nakano, Takuma Tsushio, Yoshinari Watanabe, Sara Minamikawa, Chieko Ishiguro, Kenji Yokoyama, Kenji Fujisawa, Masaya Tanahashi, Hideki Suganami, Atsushi Kasano

Supplementary Table 1. Codes used for patient inclusion — 2

Supplementary Table 2. Covariates and the codes used to select them — 3

Supplementary Table 3. Codes related to outcomes — 12

Supplementary Table 4. Demographics and other baseline characteristics — 14

Supplementary Table 5. Summary of case numbers, follow-up periods, and event occurrences in primary and secondary analysis — 17

Supplemental Figure 1. Study design diagram — 18

Supplemental Figure 2. Histogram of propensity score: ITT analysis and PP analysis — 19

Supplemental Figure 3. Kaplan-Meier plot of time to first VTE event — 21

Supplemental Figure 4. Forest plot of time to first VTE event for subgroup: ITT analysis — 23

Supplementary Table 1. Codes used for patient inclusion

Disease/Drug	ICD-10	ATC code	Prescription code
Dyslipidemia	E780, E781, E782, E783, E784, E785, E786, E788, E789		
Pemafibrate		C10A2	622573101
Fibrate (except pemafibrate)		C10A2	612180106, 612180028, 620338317, 620009426, 622039501, 622039601, 622090701, 622090801, 622096801, 622096901, 622590501, 622590601, 622892400, 622892500, 610407028, 610422259, 610422261, 610422262, 610422263, 610422264, 610422265, 610422276, 610433008, 620001883, 620002123, 620002540, 620002541, 620008508, 620339201, 620339401, 620339501, 620339603, 620339604, 620340201, 620340602, 620340603, 620340901, 620341001, 620341102, 620341301, 621254601, 622026702, 622096101, 622096102, 622223601, 622322900, 622728600, 622728700

List revised November 2024.
ATC, anatomical therapeutic chemical; ICD-10, International Statistical Classification of Diseases and Related Health Problems 10th Revision.

Supplementary Table 2. Covariates and the codes used to select them

Variable	ICD-10 or procedure/prescription code
Age	
Sex	
BMI	
Smoking history	
Hypertension	I10x-15x
Diabetes mellitus	E10x-14x
Chronic kidney disease	I12.0, N03.2-03.7, N05.2-05.7, N18.x, N19.x, N25.0, Z94.0, Z99.2
Liver disease	B18.x, K70.0-70.4, K70.9, K71.3, K71.7, K73.x, K74.x, K76.0, K76.2-76.9, Z94.4
Cancer	C00x-97x
Myocardial infarction	I21x, I22x, I25.2
Heart failure	I09.9, I11.0x, I25.5, I42.0, I42.5-42.9, I50.0x, I50.1x, I50.9x, P29.0
Atrial fibrillation/flutter	I48.x
Cerebrovascular disease	G45.x, H34.0, I60.x-69.x
Pneumonia	A01.0, A02.2, A16.2, A24.1, A40.3, A41.9, A48.1, A48.2, A49.1, A50.0, A54.8, A69.8, A70, B01.2, B05.2, B06.8, B20.6, B22.1, B25.0, B59, B77.8, C34.9, G00.1, I30.1, J02.8, J11.0, J12.0, J12.1, J12.2, J12.3, J12.8, J12.9, J13, J14, J15.0-15.9, J16.0, J18.0, J18.1, J18.2, J18.8, J18.9, J20.2, J67.2, J67.8, J67.9, J68.0, J69.0, J69.1, J69.8, J70.0, J70.4, J82, J84.1, J84.9, J85.0, J85.1, J95.8, K65.0, M00.19, M05.10, M32.1, M33.0-33.2, M35.1, O29.0, P23.0, P23.1, P23.2, P23.3, P23.4, P23.5, P23.6, P23.9, P36.1, U07.1
Chronic pulmonary disease	I27.8, I27.9, J40.x-47.x, J60.x-67.x, J68.4, J70.1, J70.3
Urinary tract infection	N10, N15.1, N30, N34, N39.0, N41, N45, N73
Connective tissue disease	M05, M06, M32, M33, M34, M35.1, M35.3, M36.0
HIV	B20-24
Crohn's disease or ulcerative colitis	K50.0, K50.1, K50.8, K50.9, K51.0, K51.2, K51.3, K51.5, K51.8, K51.9
Superficial vein thrombosis	I80.9
Varicose vein	I83.0, I83.1, I83.9
Coagulopathy	D65.x, D66.x, D67.x, D68.x, D69.x
Fracture, Trauma, Injury	S00.x-99.x, T00.x-14.x
Surgery	Class K (Surgery) excluding K9 (Transfusion) and K620 (installation or removal of IVC filter) 150000190, 150000290, 150000490, 150000590, 150000690, 150000790, 150001010, 150001110, 150001250, 150001310, 150001470, 150001570, 150012750, 150146350, 150244470, 150266670, 150268890, 150268970, 150284010, 150297990, 150306890, 150342890, 150371290, 150371390, 150371490, 150371590, 150371690, 150382490, 150383310, 150383410, 150391350, 150391650, 150391750, 150391850, 150391950, 150392550, 150392650, 150393050, 150410050, 150425190, 150306910, 150307010, 150307110, 150307310, 150307410, 150001810, 150001910, 150002010, 150002210, 150002310, 150002410, 150342970, 150368370, 150423370, 150002510, 150002610, 150283610, 150260710, 150260810, 150283710, 150003110, 150003210, 150272410, 150003510, 150003610, 150272510, 150383510, 150307710, 150307810, 150308010, 150308110, 150004050, 150004150, 150282850, 150333510, 150333610, 150004210, 150260910, 150343070, 150351910, 150411250, 150004510, 150252110, 150004610, 150004710, 150272610, 150004810, 150004910, 150005010, 150005110, 150006410,

Supplementary Table 2. Continued.

Variable	ICD-10 or procedure/prescription code
Surgery, continued.	150006510, 150006610, 150006710, 150442750, 150442850, 150443050, 150333710, 150333810, 150333910, 150334010, 150007610, 150343110, 150343210, 150343310, 150343410, 150343510, 150007810, 150007910, 150008010, 150008810, 150009010, 150371710, 150371810, 150009110, 150009210, 150009310, 150308410, 150290310, 150290410, 150371910, 150372010, 150020350, 150020450, 150009410, 150009510, 150009610, 150009710, 150009810, 150308510, 150009910, 150010010, 150010110, 150010210, 150010310, 150288510, 150010610, 150010810, 150010910, 150011010, 150011110, 150011210, 150011310, 150011410, 150308610, 150011510, 150011610, 150011710, 150011810, 150011910, 150012010, 150012110, 150308710, 150411370, 150013110, 150272910, 150010410, 150288610, 150010510, 150288710, 150300110, 150012310, 150288910, 150280050, 150013210, 150289010, 150013510, 150273010, 150013410, 150273110, 150343610, 150383610, 150013710, 150013810, 150013910, 150014010, 150014110, 150014210, 150014310, 150014410, 150014510, 150014610, 150015850, 150015950, 150016050, 150016150, 150016350, 150016450, 150016510, 150016610, 150016710, 150016810, 150016910, 150017010, 150017110, 150017210, 150017310, 150018210, 150018310, 150018410, 150018510, 150018610, 150018710, 150018810, 150018910, 150261010, 150289110, 150019010, 150019110, 150019210, 150019310, 150019410, 150019510, 150019610, 150019710, 150019810, 150261110, 150289210, 150294810, 150425670, 150352010, 150352110, 150352210, 150352310, 150352410, 150352510, 150352610, 150352710, 150395810, 150242910, 150284110, 150334110, 150020510, 150020610, 150020710, 150020810, 150020910, 150021010, 150021110, 150021210, 150021310, 150261210, 150289310, 150334210, 150334310, 150352810, 150352910, 150021410, 150021510, 150021610, 150021710, 150021810, 150021910, 150022010, 150022110, 150022210, 150261310, 150289410, 150022510, 150022610, 150022710, 150022810, 150022910, 150023010, 150023110, 150023210, 150023310, 150023910, 150024110, 150024210, 150024650, 150024710, 150024810, 150024910, 150025010, 150025110, 150025210, 150025310, 150025410, 150025510, 150261410, 150289510, 150026510, 150026610, 150026710, 150026810, 150026910, 150027010, 150027210, 150027310, 150411470, 150027610, 150027710, 150027810, 150027910, 150028010, 150028110, 150028210, 150028310, 150261510, 150289710, 150425710, 150308810, 150308910, 150028610, 150028710, 150028810, 150028910, 150029010, 150029110, 150029210, 150029310, 150029410, 150261710, 150289810, 150309010, 150353010, 150029710, 150029810, 150029910, 150030010, 150030110, 150030210, 150030310, 150030410, 150261810, 150370370, 150031410, 150031510, 150031610, 150294910, 150031710, 150295010, 150369450, 150383710, 150383810, 150392050, 150392150, 150353110, 150031910, 150032010, 150032110, 150032310, 150032510, 150032610, 150032710, 150395910, 150396010, 150052850, 150052950, 150053050, 150053150, 150053250, 150053350, 150053450, 150309110, 150309210, 150033810, 150033910, 150034010, 150034110, 150034210, 150034310, 150034410, 150034510, 150034610, 150034710, 150035210, 150035310, 150035410, 150035510, 150035610, 150035710, 150035810, 150035910, 150036010, 150036210, 150036310, 150036410, 150036510, 150036610, 150036710, 150036810, 150036910, 150037010, 150309310, 150309510, 150309810, 150309910, 150037110, 150037210, 150037310, 150037410, 150037510, 150037610, 150037710, 150037810, 150037910, 150310210, 150310310, 150310410, 150310610, 150310710, 150310810, 150310910, 150311010, 150038150, 150038250, 150038350, 150038550, 150038650, 150038750, 150038950, 150311110, 150311210, 150311310, 150311510, 150311610, 150311710, 150038050, 150312010, 150039050, 150312110, 150039310, 150039510, 150039510, 150039710, 150039910, 150312210, 150312310, 150312410, 150312610, 150312710, 150312810, 150040910, 150313110, 150261910, 150290510, 150313210, 150041010, 150041110, 150041210, 150041310, 150041710, 150041810, 150042010, 150042210, 150042310, 150042410, 150042510, 150042610, 150042710, 150042810, 150042910, 150043010, 150043110, 150043210, 150043310, 150043410, 150353210, 150353410, 150353610, 150353710, 150353810, 150353910, 150354010, 150043510, 150043610, 150043710,

Supplementary Table 2. Continued.

Variable	ICD-10 or procedure/prescription code
Surgery, continued.	150290010, 150313310, 150313410, 150313610, 150044310, 150044410, 150044510, 150044710, 150044810, 150044910, 150045010, 150045110, 150045210, 150045310, 150045410, 150045610, 150045710, 150045810, 150046010, 150396110, 150396210, 150396310, 150396510, 150396610, 150396710, 150396810, 150046110, 150046210, 150046310, 150046510, 150046610, 150046710, 150046810, 150046910, 150047010, 150047110, 150047210, 150047310, 150047410, 150047510, 150047610, 150047710, 150047810, 150047910, 150048010, 150048110, 150290210, 150313710, 150313810, 150314010, 150354110, 150411570, 150048210, 150048310, 150048410, 150048510, 150048610, 150048710, 150048810, 150048910, 150049010, 150049170, 150049250, 150343710, 150354210, 150343810, 150354310, 150425810, 150384210, 150384310, 150384410, 150425910, 150426010, 150049410, 150049510, 150049810, 150050010, 150435770, 150050310, 150050410, 150050510, 150050710, 150050810, 150050910, 150051110, 150300210, 150300310, 150300410, 150300610, 150300810, 150301010, 150255910, 150256010, 150256110, 150256310, 150256510, 150256710, 150397010, 150411610, 150411710, 150243010, 150051310, 150051410, 150051510, 150051610, 150051710, 150051810, 150052050, 150051910, 150052110, 150052210, 150052310, 150052410, 150052510, 150052610, 150052710, 150053510, 150053610, 150053710, 150053810, 150053910, 150054010, 150054110, 150054210, 150054310, 150054610, 150054710, 150055110, 150314110, 150055410, 150354510, 150354610, 150350550, 150055710, 150055810, 150055910, 150056010, 150290710, 150290810, 150290910, 150056310, 150056510, 150056850, 150057210, 150057410, 150057510, 150058310, 150058410, 150058610, 150058710, 150058810, 150058910, 150059310, 150059410, 150059510, 150059650, 150059750, 150059810, 150060210, 150060310, 150017950, 150060410, 150060810, 150384510, 150060910, 150061010, 150061110, 150061210, 150061310, 150061310, 150061410, 150061510, 150305950, 150306050, 150306150, 150341850, 150341950, 150384150, 150392350, 150369550, 150061810, 150061910, 150314210, 150062910, 150397110, 150063110, 150063210, 150063310, 150273310, 150314310, 150314410, 150410650, 150411810, 150063710, 150063810, 150063910, 150064010, 150354810, 150064210, 150064410, 150064610, 150064810, 150314510, 150354910, 150072950, 150282510, 150282610, 150314610, 150314710, 150355010, 150355110, 150368870, 150368970, 150369070, 150369170, 150369270, 150369370, 150282750, 150343910, 150344010, 150344110, 150314810, 150355210, 150397210, 150397310, 150397410, 150426110, 150066110, 150243210, 150066210, 150426210, 150067110, 150411910, 150067210, 150067410, 150335610, 150397510, 150067510, 150291010, 150067850, 150067910, 150068010, 150291110, 150291210, 150291310, 150372210, 180058750, 150068610, 150068710, 150068850, 150068910, 150069050, 150069110, 150069210, 150069410, 150069510, 150069610, 150069710, 150248250, 150273410, 150069850, 150069950, 150335710, 150372310, 150070010, 150070110, 150070210, 150070310, 150070510, 150284510, 150370470, 150372470, 150425070, 150426310, 150426410, 150071010, 150384610, 150384710, 150438470, 150071110, 150412010, 150412110, 150071310, 150301610, 150301710, 150426510, 150426610, 150426710, 150071650, 150243410, 150243510, 150243610, 150243710, 150243810, 150243910, 150344370, 150397670, 150254910, 150344410, 150355410, 150273510, 150301110, 150301210, 150372510, 150380850, 150072010, 150072110, 150072210, 150335810, 150255110, 150315010, 150291410, 150298050, 150355510, 150355610, 150412210, 150412310, 150412410, 150072310, 150273610, 150315210, 150369650, 150397710, 150397810, 150072510, 150372610, 150072610, 150072810, 150073110, 150355710, 150397910, 150426810, 150244010, 150384810, 150384970, 150398010, 150398110, 150385010, 150398210, 150315310, 150315410, 150315510, 150372710, 150398310, 150398410, 150372810, 150073210, 150073310, 150073410, 150073510, 150273710, 150256850, 150256950, 150280350, 150257050, 150257150, 150280450, 150074910, 150075010, 150273810, 150075550, 150075650, 150299650, 150299750, 150075810, 150076010, 150076310, 150295310, 150076610, 150284210, 150076710, 150076810, 150355810, 150076910, 150077010, 150077210, 150077310, 150077510,

Supplementary Table 2. Continued.

Variable	ICD-10 or procedure/prescription code
Surgery, continued.	150077610, 150077750, 150077910, 150078010, 150077810, 150078210, 150078310, 150078410, 150291610, 150078510, 150078610, 150355910, 150426910, 150078710, 150078810, 150078910, 150079010, 150079110, 150079410, 150079650, 150079510, 150079710, 150079810, 150079910, 150080050, 150080210, 150080610, 150291710, 150080750, 150427010, 150080810, 150081310, 150295410, 150081510, 150081610, 150081810, 150082110, 150082210, 150082310, 150082610, 150082710, 150083010, 150083210, 150083310, 150083410, 150083510, 150083610, 150273910, 150427110, 150083710, 150083810, 150083910, 150295510, 150084950, 150085010, 150085210, 150295710, 150085410, 150085710, 150344510, 150085810, 150085910, 150086010, 150086210, 150350150, 150412570, 150424850, 150086310, 150372910, 150086410, 150087110, 150087510, 150088410, 150335910, 150356010, 150373010, 150395150, 150427210, 150427310, 150435810, 150088610, 150088710, 150088810, 150088910, 150089010, 150089110, 150089410, 150244110, 150244210, 150248350, 150090110, 150291810, 150090210, 150090410, 150090610, 150274010, 150356110, 150252810, 150373110, 150253010, 150315610, 150356210, 150356310, 150385170, 150412670, 150280650, 150091610, 150091710, 150091810, 150091910, 150092010, 150092110, 150092210, 150092310, 150092450, 150092510, 150092610, 150092710, 150092910, 150093110, 150093210, 150274110, 150093410, 150093510, 150093610, 150093710, 150093810, 150093910, 150094010, 150094110, 150094550, 150094610, 150094710, 150094810, 150094910, 150095010, 150095210, 150095310, 150095910, 150096010, 150096110, 150398510, 150398610, 150427410, 150427510, 150096210, 150096350, 150096610, 150096910, 150255010, 150266510, 150097110, 150097210, 150097310, 150097450, 150356410, 150097710, 150097950, 150098010, 150274210, 150098450, 150098250, 150098610, 150098710, 150098810, 150344610, 150099010, 150099110, 150373210, 150373310, 150373510, 150373610, 150373710, 150099210, 150099310, 150099410, 150099510, 150427610, 150099710, 150099910, 150100110, 150292010, 150385210, 150385310, 150385410, 150385510, 150385610, 150100510, 150100710, 150100850, 150100950, 150101010, 150373810, 150101510, 150101610, 150101710, 150101810, 150101910, 150292110, 150102510, 150102710, 150102810, 150103010, 150103110, 150103310, 150103410, 150103510, 150257250, 150103810, 150103910, 150104010, 150104110, 150104210, 150412710, 150441110, 150104510, 150114450, 150104610, 150104810, 150104910, 150248450, 150248550, 150105010, 150344710, 150344810, 150344910, 150105310, 150105510, 150105610, 150105750, 150105810, 150106010, 150336010, 150106110, 150106210, 150412810, 150106310, 150106410, 150315710, 150106750, 150106850, 150299450, 150315910, 150316010, 150106910, 150107010, 150107110, 150107310, 150107450, 150107510, 150107610, 150107810, 150107910, 150412910, 150413010, 150441210, 150108110, 150108210, 150108410, 150108550, 150108610, 150108710, 150108810, 150108910, 150109010, 150109110, 150109310, 150109610, 150109710, 150109810, 150109910, 150345110, 150345210, 150345310, 150345410, 150110110, 150110210, 150110410, 150275070, 150385770, 150110610, 150316110, 150110710, 150110810, 150110910, 150345510, 150114350, 150111010, 150111110, 150111210, 150111410, 150111510, 150111710, 150111810, 150112050, 150112110, 150112210, 150253310, 150054850, 150112710, 150112810, 150296010, 150112910, 150113010, 150113110, 150113310, 150296110, 150113410, 150113610, 150114110, 150316210, 150316410, 150114510, 150274310, 150114610, 150267270, 150114710, 150274410, 150345610, 150345710, 150114810, 150114910, 150115010, 150115110, 150115210, 150115310, 150115410, 150115510, 150115610, 150115710, 150115810, 150115910, 150413110, 150116110, 150116210, 150116310, 150116410, 150116510, 150116710, 150268410, 150268510, 150268710, 150279770, 150262210, 150262310, 150117010, 150117110, 150117210, 150117310, 150274510, 150373970, 150117410, 150117510, 150117610, 150117710, 150117810, 150118110, 150118210, 150118310, 150118410, 150118610, 150118910, 150119010, 150386010, 150386110, 150119310, 150386210, 150119410, 150119510, 150413410, 150413510, 150413610, 150398810, 150398910, 150119710, 150119810, 150386310, 150119910,

Supplementary Table 2. Continued.

Variable	ICD-10 or procedure/prescription code
Surgery, continued.	150120110, 150120210, 150120410, 150262610, 150120610, 150423650, 150120910, 150121110, 150121210, 150274610, 150303010, 150399010, 150399110, 150121410, 150413710, 150121610, 150121710, 150121810, 150121910, 150262710, 150303110, 150316510, 150345870, 150345970, 150386410, 150386510, 150292210, 150292310, 150316610, 150316710, 150374010, 150122210, 150122310, 150122410, 150122710, 150123010, 150123210, 150123310, 150123610, 150296210, 150123810, 150123910, 150124150, 150124250, 150124310, 150124410, 150124510, 150336310, 150413810, 150124710, 150127350, 150356910, 150357010, 150125610, 150125910, 150427910, 150126610, 150126710, 150292410, 150316810, 150316910, 150317010, 150413910, 150127210, 150357110, 150127510, 150127610, 150127810, 150128210, 150260550, 150357210, 150128310, 150292510, 150292710, 150294410, 150294710, 150357310, 150374110, 150374210, 150414010, 150128510, 150128610, 150357410, 150374310, 150405910, 150129010, 150129110, 150281250, 150357510, 150382550, 150129210, 150234450, 150346010, 150374410, 150129410, 150281450, 150346110, 150357610, 150129710, 150129810, 150129910, 150130010, 150130110, 150266610, 150357710, 150414110, 150414210, 150414310, 150270750, 150406010, 150270850, 150298750, 150357810, 150357910, 150358010, 150358110, 150358210, 150358310, 150358410, 150358510, 150374510, 150386610, 150358610, 150358710, 150358810, 150406110, 150414410, 150428010, 150317410, 150317510, 150317670, 150414570, 150131210, 150131310, 150131610, 150131710, 150131810, 150132110, 150132210, 150132310, 150399310, 150132410, 150132610, 150132710, 150132810, 150346210, 150281350, 150346310, 150133010, 150133110, 150133210, 150399410, 150133610, 150358910, 150399510, 150133810, 150133910, 150134010, 150374610, 150253510, 150253610, 150274710, 150288310, 150317710, 150274810, 150336810, 150281550, 150382950, 150134110, 150134210, 150414710, 150386710, 150135110, 150135210, 150135310, 150328650, 150386970, 150374710, 150374810, 150387070, 150406210, 150406310, 150442510, 150399610, 150414810, 150296310, 150387110, 150135710, 150135810, 150136110, 150136210, 150366910, 150136510, 150270150, 150428310, 150136610, 150136710, 150136810, 150359110, 150137210, 150137310, 150137410, 150137810, 150137910, 150138010, 150138110, 150275110, 150138210, 150138310, 150138410, 150138510, 150359210, 150138710, 150140510, 150140610, 150140710, 150140810, 150317810, 150317910, 150318010, 150318110, 150318210, 150140010, 150374910, 150375010, 150375110, 150260350, 150284310, 150359310, 150443750, 150375210, 150375310, 150375410, 160107550, 150318310, 150145710, 150145810, 150145910, 150146010, 150302770, 150318410, 150318510, 150143010, 150143110, 150318610, 150318710, 150318810, 150318910, 150319010, 150319110, 150319210, 150319310, 150319410, 150319510, 150141010, 150279510, 150279610, 150399710, 150399810, 150406410, 150406510, 150141410, 150141610, 150141710, 150359470, 150387210, 150387310, 150399910, 150400010, 150400170, 150400270, 150260050, 150143810, 150141510, 150375570, 150375670, 150375770, 150139310, 150242550, 150400310, 150150010, 150150110, 150244910, 150245010, 150245110, 150245210, 150264810, 150275910, 150359510, 150359610, 150359710, 150359810, 150359910, 150375870, 150375970, 150381550, 150381650, 150381750, 150381850, 150381950, 150382050, 150301310, 150301410, 150301510, 150400410, 150151810, 150319710, 150139110, 150138810, 150151910, 150346410, 150320310, 150142910, 150320510, 150260150, 150346510, 150145110, 150376310, 150144910, 150139610, 150142410, 150141810, 150320610, 150411050, 150142110, 150142210, 150142810, 150144010, 150320710, 150144550, 150147410, 150147510, 150320810, 150146510, 150321110, 150376470, 150146910, 150321810, 150321910, 150376770, 150145510, 150145410, 150322010, 150144310, 150346610, 150253810, 150253910, 150275610, 150410850, 150415010, 150424650, 150400510, 150262810, 150346710, 150346870, 150370050, 150303310, 150267310, 150140110, 150140210, 150395350, 150140410, 150346910, 150347010, 150303210, 150415110, 150415210, 150322210, 150415310, 150415410, 150383250, 150387410, 150415510, 150275310, 150415610, 150415710, 150336910, 150415810,

Supplementary Table 2. Continued.

Variable	ICD-10 or procedure/prescription code
Surgery, continued.	150415910, 150337010, 150416010, 150416110, 150387510, 150387610, 150148010, 150148110, 150147610, 150147770, 150147870, 150147910, 150275870, 150347170, 150428410, 150428510, 150262910, 150275710, 150395450, 150395550, 150266110, 150266210, 150301810, 150360110, 150360210, 150360310, 150360410, 150148210, 150148310, 150148410, 150148610, 150148750, 150306550, 150148910, 150149010, 150149110, 150001650, 150148850, 150149410, 150149510, 150322710, 150337110, 150150310, 150150410, 150150510, 150150610, 150276010, 150299250, 150151150, 150151250, 150151350, 150151450, 130007970, 130008070, 130008170, 130010050, 130010150, 130010250, 130010350, 150151710, 150400610, 150400710, 150416410, 150416510, 150152010, 150152210, 150152310, 150152410, 150152510, 150152710, 150360510, 150387810, 150360610, 150360710, 150376810, 150347210, 150153810, 150157150, 150347310, 150360810, 150416610, 150416710, 150387910, 150416810, 150423050, 150423750, 150154010, 150263410, 150296510, 150428710, 150154150, 150001750, 150360910, 150376910, 150411150, 130009070, 130009170, 130009870, 150154210, 150154310, 150154430, 150154510, 150154710, 150154810, 150154910, 150155410, 150155510, 150155610, 150155710, 150293110, 150156210, 150156410, 150156510, 150156610, 150156710, 150156810, 150156910, 150322810, 150361110, 150416910, 150417010, 150417110, 150428810, 150377010, 150157510, 150157810, 150157910, 150158010, 150158210, 150158310, 150158410, 150158510, 150158610, 150158710, 150158810, 150158910, 150159010, 150159110, 150159210, 150159310, 150159410, 150251110, 150251210, 150361210, 150361310, 150388010, 150388110, 150388210, 150263610, 150159710, 150260450, 150322910, 150160010, 150347410, 150361410, 150361510, 150160110, 150160210, 150160310, 150160410, 150347510, 150160610, 150160810, 150361610, 150161110, 150161310, 150161410, 150161510, 150161610, 150361710, 150377210, 150162310, 150377310, 150245310, 150162910, 150163010, 150271650, 150400810, 150163110, 150163710, 150164110, 150377410, 150377510, 150347610, 150164210, 150164410, 150276310, 150276410, 150323010, 150417210, 150323110, 150165050, 150361810, 150428910, 150164850, 150323210, 150377610, 150377710, 150417310, 150165210, 150168010, 150347770, 150323410, 150323510, 150406610, 150406710, 150165650, 150337210, 150337310, 150377810, 150377910, 150406810, 150406910, 150378010, 150166110, 150168110, 150429010, 150323610, 150323710, 150407110, 150429110, 150170610, 150171310, 150362010, 150171510, 150171610, 150362110, 150394950, 150400910, 150401010, 150409610, 150378110, 150171810, 150171910, 150276710, 150172010, 150401110, 150172110, 150172210, 150172310, 150296610, 150276810, 150276910, 150165850, 150172410, 150254110, 150173110, 150173210, 150306650, 150388310, 150169950, 150324010, 150324110, 150324210, 150362210, 150429210, 150388410, 150388510, 150417410, 150429310, 150347810, 150347910, 150255310, 150173710, 150173910, 150174110, 150174210, 150174310, 150174550, 150362310, 150362410, 150174810, 150174910, 150362510, 150388670, 150437770, 150175310, 150437670, 150175410, 150296710, 150341450, 150417510, 150437970, 150438270, 150254410, 150437870, 150263810, 150401310, 150175610, 150175710, 150175810, 150337410, 150175910, 150277710, 150176110, 150176210, 150362610, 150362710, 150362810, 150362910, 150363010, 150363110, 150363210, 150401470, 150417610, 150417710, 150348010, 150348110, 150388710, 150388810, 150388910, 150389010, 150417810, 150417910, 150441610, 150441810, 150441910, 150442010, 150442410, 150177210, 150177310, 150177410, 150378210, 150378310, 150378410, 150378510, 150378610, 150378710, 150418070, 150443350, 150284810, 150418170, 150324310, 150324410, 150418270, 150277310, 150348210, 150177810, 150177910, 150370250, 150378870, 150348310, 150389110, 150401510, 150178110, 150178210, 150277410, 150277510, 150348410, 150389210, 150389310, 150418310, 150418410, 150178410, 150296910, 150297010, 150297110, 150297210, 150389410, 150418510, 150418610, 150418710, 150418810, 150178710, 150179010, 150409950, 150418910, 150179110, 150179210, 150179310, 150179410, 150179550, 150363610, 150179610, 150324710, 150324810, 150419370, 150179710,

Supplementary Table 2. Continued.

Variable	ICD-10 or procedure/prescription code
Surgery, continued.	150179810, 150271850, 150180010, 150180110, 150180210, 150180350, 150271550, 150180450, 150180550, 150181010, 150181110, 150389510, 150180650, 150181210, 150297310, 150299350, 150271950, 150363710, 150181310, 150181610, 150337510, 150272050, 150337610, 150180750, 150180850, 150180950, 150181710, 150181810, 150181910, 150420070, 150277810, 150337710, 150420170, 150324910, 150442110, 150363810, 150420210, 150183110, 150183510, 150297410, 150183410, 150285010, 150429570, 150437170, 150325010, 150437270, 150363910, 150437370, 150429610, 150263950, 150437470, 150184110, 150184310, 150184410, 150364010, 150184510, 150389610, 150184710, 150184810, 150184910, 150364210, 150185210, 150185310, 150402110, 150185410, 150185510, 150402210, 150185610, 150185710, 150420310, 150420410, 150420510, 150442610, 150185910, 150364310, 150293310, 150437570, 150438370, 150364410, 150186110, 150186210, 150186510, 150186610, 150186710, 150186810, 150186910, 150187010, 150348610, 150364510, 150187110, 150187210, 150245410, 150297510, 150402470, 150420610, 150438570, 150438670, 150438770, 150438870, 150439070, 150439370, 150439470, 150440570, 150440670, 150440770, 150325210, 150337810, 150337910, 150402570, 150407210, 150407310, 150407410, 150429910, 150430110, 150439570, 150439670, 150439770, 150439870, 150439970, 150440070, 150440170, 150440270, 150440370, 150440470, 150440870, 150440970, 150441070, 150187510, 150402610, 150187710, 150187810, 150187910, 150389710, 150389810, 150364610, 150188210, 150188310, 150188410, 150188510, 150188610, 150325410, 150338010, 150402710, 150188750, 150189550, 150189050, 150189610, 150189710, 150189810, 150189910, 150190010, 150190150, 150430310, 150190210, 150190310, 150190410, 150190710, 150264010, 150190910, 150191010, 150191110, 150191210, 150191310, 150191410, 150277910, 150191810, 150191910, 150192010, 150192110, 150192250, 150192310, 150279210, 150442210, 150338110, 150245510, 150245610, 150378910, 150192810, 150364710, 150193150, 150193210, 150193710, 150193810, 150194010, 150194210, 150194310, 150194410, 150194510, 150194610, 150325710, 150338210, 150194810, 150325810, 150364810, 150264110, 150195010, 150325910, 150338310, 150195210, 150326010, 150338410, 150364910, 150389910, 150430410, 150436510, 150430510, 150195610, 150420710, 150195910, 150196110, 150326110, 150420810, 150326210, 150338510, 150196310, 150196570, 150420970, 150196410, 150338610, 150421070, 150196610, 150196750, 150365010, 150390010, 150196810, 150196910, 150197010, 150303910, 150304010, 150197110, 150248950, 150264210, 150379010, 150197210, 150403070, 150197310, 150197410, 150197510, 150197810, 150197910, 150403210, 150198310, 150348710, 150198410, 150198510, 150198610, 150198710, 150198810, 150198910, 150199010, 150199150, 150199210, 150199310, 150199450, 150348810, 150430610, 150199510, 150199610, 150245810, 150200450, 150348910, 150162150, 150379110, 150379210, 150379310, 150200510, 150200610, 150245910, 150246010, 150246110, 150246210, 150365110, 150390170, 150365210, 150403310, 150403410, 150403510, 150407510, 150407610, 150407710, 150379410, 150403610, 150403710, 150403810, 150201010, 150379510, 150201110, 150403910, 150201510, 150201610, 150201710, 150404110, 150404210, 150201950, 150365310, 150202010, 150202110, 150264310, 150349010, 150202410, 150202510, 150202610, 150202710, 150202810, 150202910, 150203010, 150246310, 150246410, 150246510, 150246810, 150246910, 150204950, 150205710, 150242450, 150260250, 150205810, 150206010, 150285610, 150326310, 150365410, 150365510, 150365610, 150421110, 150206310, 150206410, 150206510, 150293610, 150206650, 150206710, 150206810, 150207110, 150207210, 150293410, 150293510, 150207310, 150207510, 150293710, 150293810, 150207910, 150264410, 150208410, 150278310, 150404310, 150208510, 150297610, 150208610, 150208710, 150254510, 150297710, 150430910, 150431010, 150208810, 150209010, 150379610, 150379710, 150276510, 150365710, 150431110, 150404410, 150431210, 150443650, 150209310, 150326510, 150338810, 150390310, 150209510, 150209610, 150210710, 150210910, 150211010, 150211110, 150211410, 150211510, 150326610, 150211650, 150211750, 150211810, 150211910,

Supplementary Table 2. Continued.

Variable	ICD-10 or procedure/prescription code
Surgery, continued.	150212010, 150212110, 150212310, 150326810, 150212410, 150212910, 150213010, 150213150, 150190550, 150190650, 150213210, 150213410, 150404610, 150213610, 150213950, 150286050, 150214510, 150326910, 150431310, 150214610, 150264510, 150293910, 150294010, 150365910, 150215110, 150215210, 150215310, 150215410, 150390410, 150421210, 150216010, 150216150, 150216510, 150002150, 150281950, 150216650, 150327010, 150216810, 150216910, 150278510, 150294110, 150294210, 150294310, 150421310, 150421410, 150421510, 150421610, 150278610, 150421710, 150421810, 150217410, 150366010, 150217510, 150272250, 150409210, 150217610, 150327210, 150217710, 150379810, 150409310, 150409810, 150218710, 150218850, 150218950, 150431510, 150431610, 150431710, 150431870, 150431970, 150219210, 150219410, 150264610, 150219710, 150264710, 150219850, 150299850, 150220010, 150270010, 150409410, 150409510, 150421910, 150422010, 150220150, 150220250, 150220450, 150268050, 150268150, 150268250, 150220710, 150282050, 150366110, 150432010, 150432170, 150432270, 150432370, 150432470, 150221110, 150221210, 150221310, 150221410, 150221510, 150221610, 150221710, 150221810, 150221910, 150222010, 150222110, 150222210, 150390570
Blood transfusion	150224810, 150224910, 150225010, 150225110, 150225210, 150225770, 150247010, 150254810, 150286210, 150286310, 150286410, 150286510, 150327910, 150328010, 150366570, 150366670, 150380070, 150390610, 150390710, 150422110
Central venous line	130004410, 130004670, 130011610
Intravenous catheter or lead	130010050, 130010150, 130010250, 170012410

Drug	ATC code or procedure/prescription code
Statin	C10A1, C10C0, C11A1
Ezetimibe	620004868, 622704001, 622704101, 622784901, 622786001, 622789601, 622789701, 622790901, 622791901, 622792901, 622794301, 622795201, 622797701, 622799601, 622801101, 622805001, 622806401, 622808201, 622809701, 622812801, 622814501, 622846700, 622584101, 622584201, 622944001, 622676701, 622676801, 622927801, 622927901
IPE	610421343, 610421344, 610422055, 610422056, 610422123, 610422206, 610422290, 613390006, 620001924, 620005032, 620005513, 620005932, 620007887, 620007888, 620007889, 620007890, 620007891, 620007892, 620007893, 620007894, 620007895, 620007896, 620007897, 620007898, 620007993, 620007994, 620007995, 620008112, 620008113, 620815302, 620815601, 620815903, 620816201, 620816301, 620816501, 620816805, 620817001, 620817103, 620817504, 620817702, 621315603, 621776502, 621869505, 621869605, 621869705, 621959901, 621959902, 621960001, 621960002, 621960101, 621960102, 622468501, 622468601, 622468701, 622610500, 622738900, 622739000, 622739100, 622920201
ω-3 fatty acid	622198801, 622886101, 622908201, 622910601, 622913601, 622917901
Insulin	A10C, A10D0
Metformin	A10J1, A10K3, A10N3
Sulfonylurea	A10H
α-glucosidase inhibitor	A10L
SGLT2 inhibitor	A10P1, A10P5
DPP4 inhibitor	A10N1, A10N3, A10N9, A10P5

Supplementary Table 2. Continued.

Variable	ICD-10 or procedure/prescription code
GLP-1 agonist	A10S
ACE inhibitor, ARB	C09A0, C09C0, C09D1, C09D3, C09D9, C09X0
β blocker	C07A0
Ca channel blocker	C08A0, C11A1
Chemotherapy	L01x-04x
Hormone replacement therapy	628710002, 620006565, 620532301, 620532401, 620008374, 620007376, 620532406, 620006265, 620006800, 612470079, 612470080, 612470081, 610441034, 620004457, 612470049, 612470002, 612470003, 612470041, 612470033, 620534001, 620007485, 620006314, 620006313, 620008396, 620006209, 622437601, 622437701, 622437801, 620006392, 620005832, 622159201, 620004549, 620005136, 622703901, 620005893, 622368101, 620008653, 612470029, 622877501, 612470030, 612470038, 620537901, 620535801, 620006308, 620536204, 620536301, 620008391, 620007486, 642470088, 628774301, 628772801, 628775301, 628775801, 620539001, 622310100, 610407255, 610407418, 610407449, 622908401, 622534301, 622561301, 622543201, 622570801, 622545101, 622538101, 622553301, 622556601, 622565001, 622541401, 622612200, 622583601, 622580801, 622545201, 622640301, 622556701, 610454075, 610454076, 610412174, 621285301, 620537802, 620538201, 620006307, 620007479, 620540601, 642480003, 620008557, 620541401, 622534101, 621982201, 621829201, 622264001, 620540801, 620541001, 620007058, 622646201, 620541801, 642470048, 620008569, 622915701, 622452201, 622660801, 622588401, 622657301, 622452202, 622662401, 622655701, 622657401, 622647401, 620005958, 612490044, 620551901, 622515601, 620514304, 622916201, 620514100, 622916001, 620514201, 622916101, 642410032, 620001308, 620001309, 620514306, 620006207, 620006205, 620006206, 620007469, 620004863, 620007470, 620004864, 622017201, 620009107, 620009108, 620003473, 620003472, 629913801, 629913901, 629914001, 621809004, 621809104, 640453035, 640453034, 620513701, 622184601, 620513301, 620008172, 620513404, 620513004, 620513402, 620007220, 620513002, 620007219, 620002944, 620002943, 620000281, 640454039, 621698703, 622915901, 621698603, 622915801, 622003201, 620001904, 622811701, 622520602, 622458301, 622495301, 622514501, 622568701, 622554801, 622532201, 622503101, 622498201, 622520601, 610407429, 610407430, 643250061
Platelet aggregation inhibitor	B01Cx
Thrombolytic	643950056, 643950057, 643950058, 643950059, 643950060, 643950061, 640463027, 640463026, 620006202, 620006203, 620006204, 620007270, 620007271, 620007272
Oral anticoagulant	620002332, 613330003, 613330004, 622122601, 621938101, 621940901, 621480509, 621480507, 621480504, 621480506, 610450012, 620811502, 620811511, 620811507, 620811510, 620811503, 621480604, 610463227, 613330001, 610463228, 613330002, 610462024, 620000731, 610462025, 622043301, 622043401, 622224901, 622225001, 622576001, 622576101, 622576201, 622080901, 622081001, 622375201, 622829001, 622829101, 622853901, 622449101, 622449201, 622919801, 622068301, 622068401
Parenteral anticoagulation	621824802, 621824902, 621825102, 622458001, 620812203, 621824702, 621825002, 621933401, 620006726, 621825502, 620008392, 621825302, 621825802, 621825704, 621826102, 621826004, 621826402, 621825602, 620812504, 620006725, 643330011, 620006728, 620006734, 620006739, 620006786, 620004874, 620004875, 622044501, 622044601

List revised November 2024.
ACE, angiotensin converting enzyme; ARB, angiotensin II receptor blocker; ATC, anatomical therapeutic chemical; BMI, body mass index; DPP4, dipeptidyl peptidase-4; GLP-1, glucagon-like peptide-1; HIV, human immunodeficiency virus;
ICD-10, International Statistical Classification of Diseases and Related Health Problems 10th Revision; IPE, icosapent ethyl;
IVC, inferior vena cava; SGLT2, sodium glucose cotransporter 2.

Supplementary Table 3. Codes related to outcomes

Disease	PE/DVT	ICD-10 (diagnosis code)
Acute pulmonary heart	PE	I260
Idiopathic chronic pulmonary thromboembolism	PE	I269
Pulmonary thromboembolism	PE	I269
Pulmonary infarction	PE	I269
Pulmonary embolism	PE	I269
Pulmonary vein thromboembolism	PE	I269
Pulmonary vein thrombosis	PE	I269
Pulmonary artery thromboembolism	PE	I269
Pulmonary artery thrombosis	PE	I269
Chronic pulmonary thromboembolism	PE	I269
Thrombophlebitis of the lower extremities	DVT	I800
Leg thrombophlebitis	DVT	I800
Femoral thrombophlebitis	DVT	I801
Femoral phlebitis	DVT	I801
Femoral vein thrombosis	DVT	I801
Venous thrombosis in the lower extremities	DVT	I802
Varicose vein thrombosis sequelae	DVT	I802
Venous thrombosis in the lower leg	DVT	I802
Deep vein thrombosis	DVT	I802
Venous inflammation of the lower limbs	DVT	I803
Leg phlebitis	DVT	I803
Leg thrombophlebitis	DVT	I803
Mondor’s disease	DVT	I808
Lemierre’s syndrome	DVT	I808
Dorsal phlebitis	DVT	I808
Thrombophlebitis of the upper extremities	DVT	I808
Varicose veins in the upper extremities	DVT	I808
Thrombophlebitis of the upper arm	DVT	I808
Brachial phlebitis	DVT	I808
Esophageal phlebitis	DVT	I808
Thrombophlebitis of the forearm	DVT	I808
Phlebitis forearm	DVT	I808
Pyogenic phlebitis	DVT	I809
Thrombophlebitis	DVT	I809
Phlebitis	DVT	I809
Perivenous inflammation	DVT	I809
Endo phlebitis	DVT	I809

Supplementary Table 3. Continued.

Disease	PE/DVT	ICD-10 (diagnosis code)
Superficial phlebitis	DVT	I809
Budd-Chiari syndrome	DVT	I820
Hepatic venous thrombosis	DVT	I820
Hepatic vein embolism	DVT	I820
Migratory thrombophlebitis	DVT	I821
Inferior vena cava thrombosis	DVT	I822
Vena cava embolism	DVT	I822
Renal vein thrombosis	DVT	I823
Renal vein embolism	DVT	I823
Venous thrombosis in the upper extremities	DVT	I828
Brachial vein thrombosis	DVT	I828
Thrombosis of the veins of the forearm	DVT	I828
Iliac vein compression syndrome	DVT	I828
Acute venous thrombosis	DVT	I829
Venous thrombosis	DVT	I829
Venous embolism	DVT	I829
Idiopathic thrombosis due to hereditary thrombophilia predisposition	DVT	I829, D689 (8849245)

Drug	Prescription code
Thrombolytic	640463027, 640463026, 620006202, 620006203, 620006204, 620007270, 620007271, 620007272
Oral anticoagulant	620002332, 613330003, 613330004, 622122601, 621938101, 621940901, 621480509, 621480507, 621480504, 621480506, 610450012, 620811502, 620811511, 620811507, 620811510, 620811503, 621480604, 610463227, 613330001, 610463228, 613330002, 610462024, 620000731, 610462025, 622043301, 622043401, 622224901, 622225001, 622576001, 622576101, 622576201, 622080901, 622081001, 622375201, 622829001, 622829101, 622853901, 622449101, 622449201, 622919801, 622068301, 622068401
Parenteral anticoagulant	621824802, 621824902, 621825102, 622458001, 620812203, 621824702, 621825002, 621933401, 620006726, 621825502, 620008392, 621825302, 621825802 ,621825704, 621826102, 621826004, 621826402, 621825602, 620812504, 620006725, 643330011, 620006728, 620006734, 620006739, 620004874, 620004875, 622044501, 622044601

List revised November 2024.
DVT, deep vein thrombosis; ICD-10, International Statistical Classification of Diseases and Related Health Problems, 10th Revision; PE, pulmonary embolism.

Supplementary Table 4. Demographics and other baseline characteristics

		Total N=139,170	Exposed group N=23,195	Control group N=115,975	StdDiff	After MI and SMRW				
						StdDiff: ITT (min, max)	StdDiff: PP gap or grace period = 0 days (min, max)	StdDiff: PP gap or grace period = 30 days (min, max)	StdDiff: PP gap or grace period = 90 days (min, max)	StdDiff: PP gap or grace period = 180 days (min, max)
Demographics										
Age, years	Median (IQR)	70.0 (58.0, 78.0)	61.0 (51.0, 71.0)	71.0 (60.0, 79.0)						
	Mean±SD	67.5±14.3	60.6±13.9	68.9±14.0						
Age category	<Median	67,552 (48.5)	16,158 (69.7)	51,394 (44.3)						
	Median≤	71,618 (51.5)	7037 (30.3)	64,581 (55.7)						
Age category	20≤, <30	1391 (1.0)	383 (1.7)	1008 (0.9)	0.070	0.008 (0.008, 0.008)	0.008 (0.008, 0.009)	0.008 (0.008, 0.008)	0.008 (0.008, 0.009)	0.008 (0.008, 0.008)
	30≤, <40	4255 (3.1)	1302 (5.6)	2953 (2.5)	0.156	0.005 (0.005, 0.006)	0.005 (0.005, 0.006)	0.005 (0.005, 0.006)	0.005 (0.005, 0.006)	0.005 (0.005, 0.006)
	40≤, <50	11,762 (8.5)	3484 (15.0)	8278 (7.1)	0.253	0.000 (0.000, 0.001)	0.000 (–0.001, 0.001)	0.000 (0.000, 0.001)	0.000 (–0.001, 0.000)	0.000 (0.000, 0.001)
	50≤, <60	20,916 (15.0)	5467 (23.6)	15,449 (13.3)	0.267	–0.006 (–0.006, –0.005)	–0.006 (–0.006, –0.005)	–0.006 (–0.006, –0.005)	–0.006 (–0.006, –0.005)	–0.006 (–0.006, –0.005)
	60≤, <70	29,228 (21.0)	5522 (23.8)	23,706 (20.4)	0.081	0.001 (0.001, 0.002)	0.001 (0.000, 0.001)	0.001 (0.001, 0.002)	0.001 (0.001, 0.002)	0.001 (0.001, 0.002)
	70≤, <80	42,199 (30.3)	5212 (22.5)	36,987 (31.9)	–0.213	–0.002 (–0.003, –0.002)	–0.002 (–0.003, –0.002)	–0.002 (–0.003, –0.002)	–0.002 (–0.003, –0.002)	–0.002 (–0.003, –0.002)
	80≤, <90	25,119 (18.0)	1705 (7.4)	23,414 (20.2)	–0.379	0.002 (0.002, 0.002)	0.002 (0.002, 0.002)	0.002 (0.002, 0.002)	0.002 (0.002, 0.002)	0.002 (0.002, 0.002)
	90≤, <100	4232 (3.0)	118 (0.5)	4114 (3.5)	–0.217	0.001 (0.001, 0.001)	0.001 (0.001, 0.001)	0.001 (0.001, 0.001)	0.001 (0.001, 0.001)	0.001 (0.001, 0.001)
	100≤	68 (0.0)	2 (0.0)	66 (0.1)	–0.027	0.000 (0.000, 0.000)	0.000 (0.000, 0.000)	0.000 (0.000, 0.000)	0.000 (0.000, 0.000)	0.000 (0.000, 0.000)
Sex	Female	64,808 (46.6)	7755 (33.4)	57,053 (49.2)	0.324	–0.006 (–0.006, –0.006)	–0.006 (–0.007, –0.006)	–0.006 (–0.006, –0.006)	–0.006 (–0.007, –0.006)	–0.006 (–0.007, –0.006)
BMI, kg/m^2 [a]	Median (IQR)	23.6 (21.1, 26.3)	25.0 (22.7, 27.9)	23.3 (20.8, 25.9)						
	Mean±SD	24.0±4.3	25.6±4.4	23.6±4.2						
BMI category 1[a]	<25	15,155 (64.6)	2163 (50.2)	12,992 (67.8)						
	25≤	8314 (35.4)	2148 (49.8)	6166 (32.2)						
BMI Category 2[a]	<18.5	1870 (8.0)	122 (2.8)	1748 (9.1)	–0.268	0.004 (0.001, 0.006)	0.005 (0.003, 0.007)	0.006 (0.004, 0.008)	0.005 (0.003, 0.009)	0.005 (0.003, 0.007)
	18.5≤, <25	13,285 (56.6)	2041 (47.3)	11,244 (58.7)	–0.229	0.008 (0.005, 0.012)	0.009 (0.005, 0.012)	0.008 (0.003, 0.012)	0.009 (0.005, 0.012)	0.008 (0.004, 0.012)
	25≤, <30	6376 (27.2)	1521 (35.3)	4855 (25.3)	0.218	–0.005 (–0.010, –0.001)	–0.005 (–0.008, –0.002)	–0.006 (–0.011, 0.001)	–0.006 (–0.009, –0.003)	–0.006 (–0.009, 0.001)
	30≤, <35	1529 (6.5)	501 (11.6)	1028 (5.4)	0.226	–0.005 (–0.010, 0.000)	–0.007 (–0.012, 0.000)	–0.006 (–0.011, –0.002)	–0.006 (–0.010, –0.001)	–0.007 (–0.012, –0.002)
	35≤, <40	318 (1.4)	100 (2.3)	218 (1.1)	0.091	–0.002 (–0.008, 0.004)	–0.003 (–0.008, 0.004)	–0.002 (–0.009, 0.003)	–0.003 (–0.012, 0.001)	–0.001 (–0.007, 0.004)
	40≤	91 (0.4)	26 (0.6)	65 (0.3)	0.039	–0.003 (–0.009, 0.001)	–0.004 (–0.010, 0.003)	–0.003 (–0.007, 0.001)	–0.003 (–0.014, 0.003)	–0.003 (–0.008, 0.004)
Smoking history[b]		8665 (39.6)	2048 (51.8)	6617 (36.9)	–0.304	0.004 (0.001, 0.009)	0.005 (0.001, 0.008)	0.005 (0.002, 0.009)	0.006 (0.002, 0.010)	0.005 (–0.001, 0.009)

Supplementary Table 4. Continued.

	Total N=139,170	Exposed group N=23,195	Control group N=115,975	StdDiff	After MI and SMRW				
					StdDiff: ITT (min, max)	StdDiff: PP gap or grace period = 0 days (min, max)	StdDiff: PP gap or grace period = 30 days (min, max)	StdDiff: PP gap or grace period = 90 days (min, max)	StdDiff: PP gap or grace period = 180 days (min, max)
Medical history									
Hypertension	19,397 (13.9)	3899 (16.8)	15,498 (13.4)	−0.096	0.044 (0.043, 0.044)	0.044 (0.043, 0.044)	0.044 (0.043, 0.044)	0.044 (0.043, 0.044)	0.044 (0.043, 0.044)
Diabetes mellitus	35,434 (25.5)	8096 (34.9)	27,338 (23.6)	−0.251	0.038 (0.037, 0.038)	0.037 (0.037, 0.038)	0.037 (0.037, 0.038)	0.037 (0.037, 0.038)	0.037 (0.037, 0.038)
Chronic kidney disease	4219 (3.0)	599 (2.6)	3620 (3.1)	0.032	0.010 (0.010, 0.010)	0.010 (0.010, 0.010)	0.010 (0.009, 0.010)	0.010 (0.010, 0.010)	0.010 (0.010, 0.010)
Liver disease	11,516 (8.3)	3086 (13.3)	8430 (7.3)	−0.200	0.023 (0.023, 0.024)	0.023 (0.023, 0.024)	0.023 (0.023, 0.024)	0.023 (0.023, 0.024)	0.024 (0.023, 0.024)
Cancer	27,349 (19.7)	5008 (21.6)	22,341 (19.3)	−0.058	0.014 (0.013, 0.014)	0.014 (0.013, 0.014)	0.013 (0.013, 0.014)	0.014 (0.013, 0.014)	0.014 (0.013, 0.014)
Myocardial infarction	6364 (4.6)	1111 (4.8)	5253 (4.5)	−0.012	0.019 (0.018, 0.019)	0.019 (0.019, 0.019)	0.019 (0.018, 0.019)	0.019 (0.018, 0.019)	0.019 (0.018, 0.019)
Heart failure	17,403 (12.5)	2986 (12.9)	14,417 (12.4)	−0.013	0.019 (0.019, 0.020)	0.019 (0.019, 0.020)	0.019 (0.019, 0.020)	0.019 (0.019, 0.020)	0.019 (0.019, 0.020)
Atrial fibrillation/flutter	3927 (2.8)	563 (2.4)	3364 (2.9)	0.029	0.008 (0.007, 0.008)	0.008 (0.007, 0.008)	0.008 (0.007, 0.008)	0.008 (0.007, 0.008)	0.008 (0.007, 0.008)
Cerebrovascular disease	11,342 (8.1)	1721 (7.4)	9621 (8.3)	0.033	0.012 (0.012, 0.013)	0.012 (0.012, 0.013)	0.012 (0.012, 0.013)	0.012 (0.012, 0.013)	0.012 (0.012, 0.013)
Pneumonia	21,341 (15.3)	4163 (17.9)	17,178 (14.8)	−0.085	0.033 (0.033, 0.033)	0.033 (0.033, 0.033)	0.033 (0.032, 0.033)	0.033 (0.033, 0.034)	0.033 (0.033, 0.033)
Chronic pulmonary disease	4994 (3.6)	816 (3.5)	4178 (3.6)	0.005	0.009 (0.009, 0.009)	0.009 (0.008, 0.009)	0.009 (0.008, 0.009)	0.009 (0.009, 0.009)	0.009 (0.009, 0.009)
Urinary tract infection	7658 (5.5)	1462 (6.3)	6196 (5.3)	−0.041	0.010 (0.010, 0.010)	0.010 (0.009, 0.010)	0.010 (0.009, 0.010)	0.010 (0.010, 0.010)	0.010 (0.009, 0.010)
Connective tissue disease	4058 (2.9)	815 (3.5)	3243 (2.8)	−0.041	0.006 (0.006, 0.006)	0.006 (0.006, 0.006)	0.006 (0.005, 0.006)	0.006 (0.006, 0.006)	0.006 (0.006, 0.006)
HIV	629 (0.5)	97 (0.4)	532 (0.5)	0.006	0.001 (0.001, 0.001)	0.001 (0.001, 0.001)	0.001 (0.001, 0.001)	0.001 (0.001, 0.001)	0.001 (0.001, 0.001)
Crohn’s disease or ulcerative colitis	148 (0.1)	33 (0.1)	115 (0.1)	−0.012	−0.002 (−0.002, −0.001)	−0.002 (−0.002, −0.002)	−0.002 (−0.002, −0.001)	−0.002 (−0.002, −0.001)	−0.002 (−0.002, −0.001)
Superficial vein thrombosis	0 (0.0)	0 (0.0)	0 (0.0)	NA	NA	NA	NA	NA	NA
Varicose vein	242 (0.2)	29 (0.1)	213 (0.2)	0.015	0.003 (0.002, 0.003)	0.003 (0.002, 0.003)	0.002 (0.002, 0.003)	0.002 (0.002, 0.003)	0.002 (0.002, 0.003)
Coagulopathy	9355 (6.7)	1621 (7.0)	7734 (6.7)	−0.013	0.011 (0.010, 0.011)	0.011 (0.010, 0.011)	0.011 (0.010, 0.011)	0.011 (0.010, 0.011)	0.011 (0.010, 0.011)
Fracture, trauma, injury	6390 (4.6)	940 (4.1)	5450 (4.7)	0.032	0.008 (0.008, 0.008)	0.008 (0.007, 0.008)	0.008 (0.008, 0.008)	0.008 (0.008, 0.008)	0.008 (0.008, 0.008)
Surgery	13,319 (9.6)	2093 (9.0)	11,226 (9.7)	0.023	0.019 (0.019, 0.019)	0.019 (0.019, 0.019)	0.019 (0.019, 0.019)	0.019 (0.019, 0.019)	0.019 (0.019, 0.019)
Blood transfusion	1787 (1.3)	193 (0.8)	1594 (1.4)	0.052	0.005 (0.005, 0.005)	0.005 (0.005, 0.005)	0.005 (0.005, 0.005)	0.005 (0.005, 0.005)	0.005 (0.005, 0.005)
Central venous line	1109 (0.8)	164 (0.7)	945 (0.8)	0.012	0.010 (0.010, 0.010)	0.010 (0.010, 0.010)	0.010 (0.010, 0.010)	0.010 (0.010, 0.010)	0.010 (0.010, 0.010)
Intravenous catheter or lead	187 (0.1)	26 (0.1)	161 (0.1)	0.008	0.001 (0.000, 0.001)	0.001 (0.000, 0.001)	0.001 (0.000, 0.001)	0.001 (0.000, 0.001)	0.001 (0.000, 0.001)

Supplementary Table 4. Continued.

	Total N=139,170	Exposed group N=23,195	Control group N=115,975	StdDiff	After MI and SMRW				
					StdDiff: ITT (min, max)	StdDiff: PP gap or grace period = 0 days (min, max)	StdDiff: PP gap or grace period = 30 days (min, max)	StdDiff: PP gap or grace period = 90 days (min, max)	StdDiff: PP gap or grace period = 180 days (min, max)
Concomitant use									
Statin	34,731 (25.0)	5831 (25.1)	28,900 (24.9)	−0.005	0.030 (0.030, 0.031)	0.030 (0.030, 0.031)	0.030 (0.030, 0.031)	0.030 (0.030, 0.031)	0.030 (0.030, 0.031)
Ezetimibe	4629 (3.3)	1487 (6.4)	3142 (2.7)	−0.178	0.027 (0.026, 0.027)	0.027 (0.026, 0.027)	0.027 (0.025, 0.027)	0.027 (0.026, 0.027)	0.027 (0.026, 0.027)
IPE	2349 (1.7)	759 (3.3)	1590 (1.4)	−0.127	0.013 (0.012, 0.013)	0.012 (0.012, 0.013)	0.013 (0.012, 0.014)	0.013 (0.012, 0.013)	0.013 (0.012, 0.013)
ω-3 fatty acid	1217 (0.9)	543 (2.3)	674 (0.6)	−0.147	0.014 (0.013, 0.015)	0.014 (0.014, 0.015)	0.014 (0.013, 0.015)	0.014 (0.014, 0.015)	0.014 (0.014, 0.016)
Insulin	7123 (5.1)	2063 (8.9)	5060 (4.4)	−0.183	0.037 (0.037, 0.037)	0.037 (0.036, 0.038)	0.037 (0.036, 0.037)	0.037 (0.036, 0.038)	0.037 (0.036, 0.037)
Metformin	9193 (6.6)	3613 (15.6)	5580 (4.8)	−0.362	0.042 (0.041, 0.043)	0.043 (0.042, 0.044)	0.042 (0.042, 0.043)	0.042 (0.042, 0.043)	0.042 (0.041, 0.043)
Sulfonylurea	2617 (1.9)	753 (3.2)	1864 (1.6)	−0.107	0.015 (0.014, 0.015)	0.015 (0.014, 0.015)	0.015 (0.014, 0.015)	0.015 (0.014, 0.015)	0.015 (0.014, 0.015)
α-glucosidase inhibitor	2101 (1.5)	457 (2.0)	1644 (1.4)	−0.043	0.004 (0.004, 0.005)	0.004 (0.004, 0.005)	0.004 (0.004, 0.005)	0.004 (0.004, 0.005)	0.004 (0.004, 0.005)
SGLT2 inhibitor	8553 (6.1)	3566 (15.4)	4987 (4.3)	−0.378	0.042 (0.042, 0.043)	0.042 (0.041, 0.044)	0.043 (0.042, 0.043)	0.043 (0.042, 0.044)	0.042 (0.042, 0.043)
DPP4 inhibitor	12,661 (9.1)	3612 (15.6)	9049 (7.8)	−0.244	0.038 (0.038, 0.039)	0.039 (0.038, 0.039)	0.039 (0.038, 0.039)	0.039 (0.038, 0.039)	0.039 (0.038, 0.039)
GLP-1 agonist	2132 (1.5)	971 (4.2)	1161 (1.0)	−0.201	0.032 (0.031, 0.033)	0.032 (0.031, 0.034)	0.032 (0.031, 0.033)	0.032 (0.031, 0.033)	0.032 (0.031, 0.033)
ACE inhibitor/ARB	27,125 (19.5)	6393 (27.6)	20,732 (17.9)	−0.233	0.038 (0.038, 0.039)	0.038 (0.038, 0.039)	0.038 (0.038, 0.039)	0.038 (0.038, 0.039)	0.038 (0.038, 0.039)
β blocker	15,970 (11.5)	3466 (14.9)	12,504 (10.8)	−0.125	0.032 (0.031, 0.032)	0.032 (0.031, 0.032)	0.032 (0.031, 0.032)	0.032 (0.031, 0.032)	0.032 (0.031, 0.032)
Ca channel blocker	25,909 (18.6)	5177 (22.3)	20,732 (17.9)	−0.111	0.028 (0.028, 0.028)	0.028 (0.028, 0.029)	0.028 (0.028, 0.029)	0.028 (0.028, 0.029)	0.028 (0.028, 0.029)
Chemotherapy	6750 (4.9)	1064 (4.6)	5686 (4.9)	0.015	0.007 (0.006, 0.007)	0.007 (0.006, 0.007)	0.007 (0.006, 0.007)	0.007 (0.006, 0.007)	0.007 (0.006, 0.007)
Hormone replacement therapy	881 (0.6)	178 (0.8)	703 (0.6)	−0.020	0.001 (0.001, 0.001)	0.001 (0.001, 0.001)	0.001 (0.001, 0.001)	0.001 (0.001, 0.001)	0.001 (0.001, 0.001)
Platelet aggregation inhibitor	19,487 (14.0)	3205 (13.8)	16,282 (14.0)	0.006	0.032 (0.032, 0.033)	0.032 (0.032, 0.032)	0.032 (0.031, 0.033)	0.032 (0.032, 0.033)	0.032 (0.032, 0.033)
Thrombolytic	108 (0.1)	12 (0.1)	96 (0.1)	0.012	0.000 (0.000, 0.000)	0.000 (0.000, 0.000)	0.000 (0.000, 0.000)	0.000 (0.000, 0.000)	0.000 (−0.001, 0.000)
Oral anticoagulant	7488 (5.4)	1102 (4.8)	6386 (5.5)	0.034	0.008 (0.008, 0.009)	0.008 (0.008, 0.009)	0.008 (0.008, 0.009)	0.008 (0.008, 0.009)	0.008 (0.008, 0.009)
Parenteral anticoagulation	7971 (5.7)	1315 (5.7)	6656 (5.7)	0.003	0.020 (0.019, 0.020)	0.019 (0.019, 0.020)	0.020 (0.019, 0.020)	0.020 (0.019, 0.020)	0.020 (0.019, 0.020)

Data are given as n (%).
[a] Total N=23,469; Exposed group N=4311; Control group N=19,158.
[b] Total N=21,879; Exposed group N=3952; Control group N=17,927.
ACE, angiotensin converting enzyme; ARB, angiotensin II receptor blocker; BMI, body mass index; CI, confidence interval; DPP4, dipeptidyl peptidase-4; GLP-1, glucagon-like peptide-1; HIV, human immunodeficiency virus; IPE, icosapent ethyl; IQR, interquartile range; ITT, intention-to-treat; MI, multiple imputation; NA, not available; PP, per-protocol; SD, standard deviation; SGLT2, sodium glucose cotransporter 2; SMRW, standardized mortality ratio weight; StdDiff, standardized differences.

Supplementary Table 5. Summary of case numbers, follow-up periods, and event occurrences in primary and secondary analysis

	ITT		PP							
			gap or grace period = 0 days		gap or grace period = 30 days		gap or grace period = 90 days		gap or grace period = 180 days	
	Exposed group	Control group	Exposed group	Control group	Exposed group	Control group	Exposed group	Control group	Exposed group	Control group
N	23,195	115,975	23,195	115,975	23,195	115,975	23,195	115,975	23,195	115,975
Number of events (%)	286 (1.2)	2297 (2.0)	147 (0.6)	2239 (1.9)	199 (0.9)	2239 (1.9)	217 (0.9)	2239 (1.9)	223 (1.0)	2239 (1.9)
Average time of follow-up, years	1.30	1.49	0.51	1.44	0.82	1.44	0.91	1.44	0.95	1.44
Total time of follow-up, years	30,200.12	172,359.76	11,787.00	167,164.44	18,911.80	167,164.44	21,223.13	167,164.44	21,932.96	167,164.44
Rate of occurrence, 100 person-years	0.95	1.33	1.25	1.34	1.05	1.34	1.02	1.34	1.02	1.34
Cox Proportional Hazard Model with SMRW HR (95%CI)	0.925 (0.809 – 1.058)		0.816 (0.686 – 0.970)		0.857 (0.736 – 0.998)		0.875 (0.755 – 1.015)		0.882 (0.762 – 1.021)	

CI, confidence interval; HR, hazard ratio; ITT, intention-to-treat; PP, per-protocol; SMRW, standardized mortality ratio weight.

Supplemental Figure 1. Study design diagram

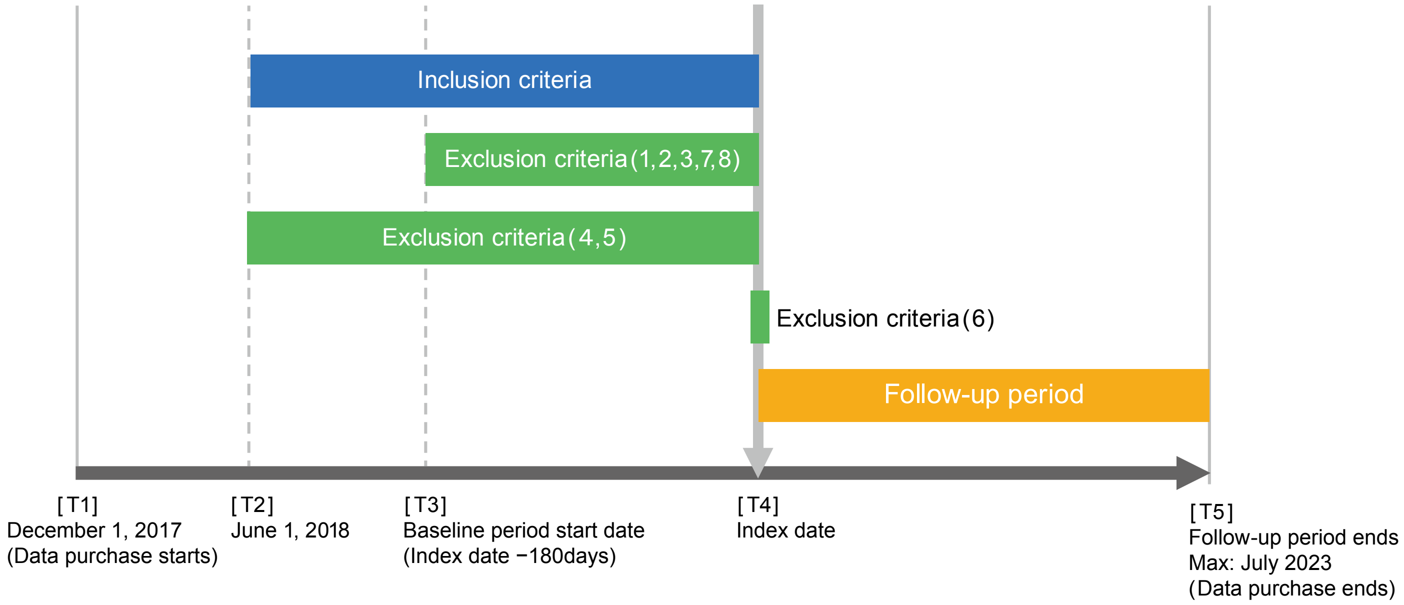

See Table 1 for exclusion criteria (1-8).

Supplemental Figure 2. Histogram of propensity score: ITT analysis and PP analysis

A Before applying SMRW method:
ITT analysis

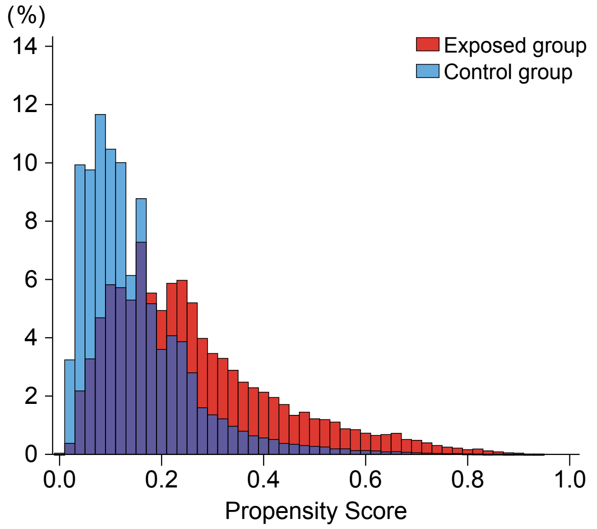

B After applying SMRW method:
ITT analysis

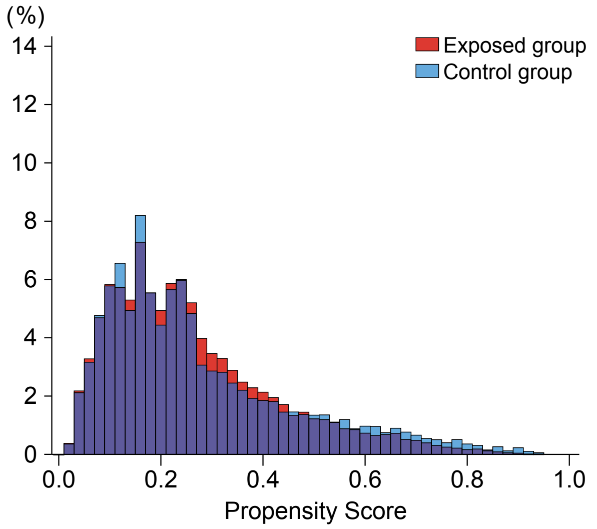

C Before applying SMRW method:
PP analysis (gap or grace period for PP analysis is 0 days)

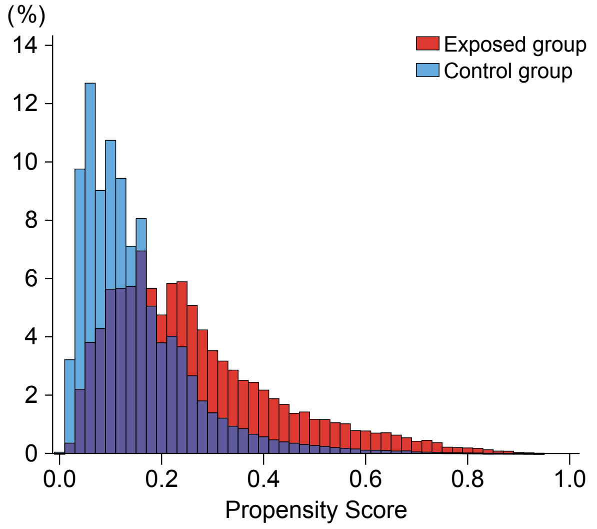

D After applying SMRW method:
PP analysis (gap or grace period for PP analysis is 0 days)

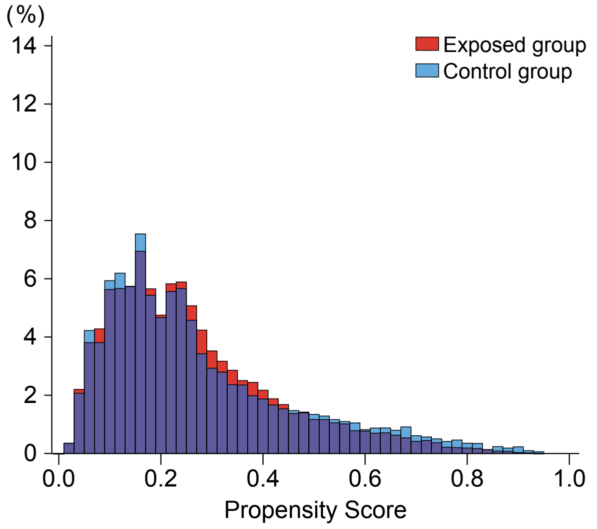

E Before applying SMRW method:
PP analysis (gap or grace period for PP analysis is 30 days)

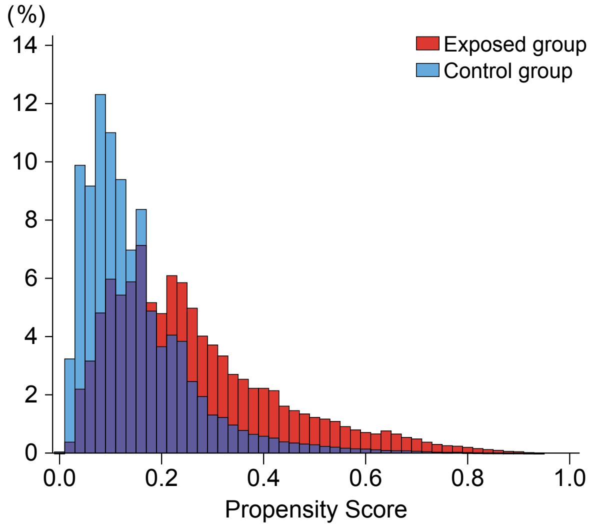

F After applying SMRW method:
PP analysis (gap or grace period for PP analysis is 30 days)

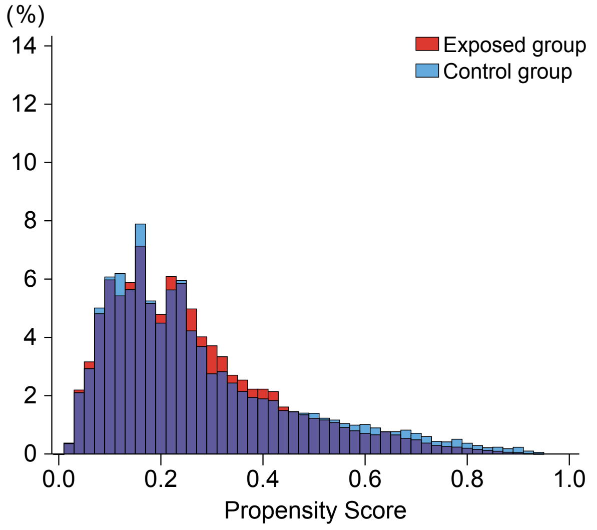

Supplemental Figure 2. Continued.

G Before applying SMRW method:
PP analysis (gap or grace period for PP analysis is 90 days)

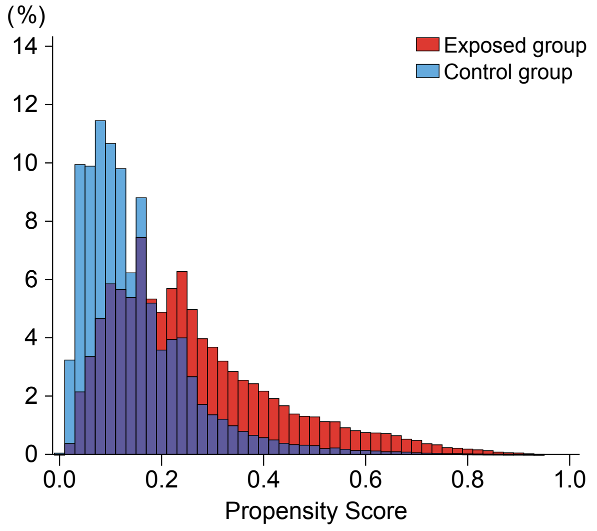

H After applying SMRW method:
PP analysis (gap or grace period for PP analysis is 90 days)

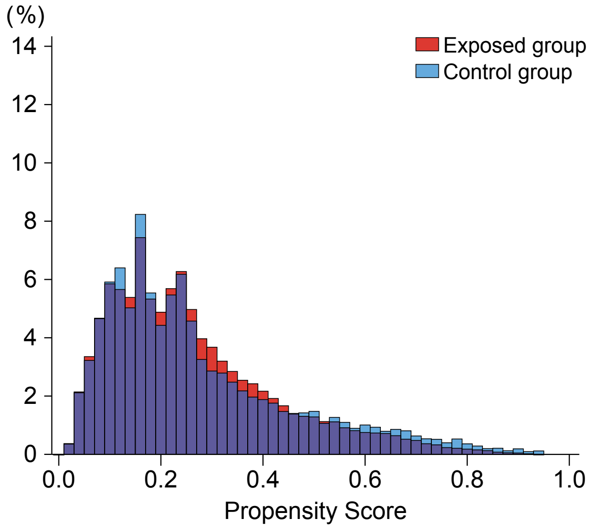

I Before applying SMRW method:
PP analysis (gap or grace period for PP analysis is 180 days)

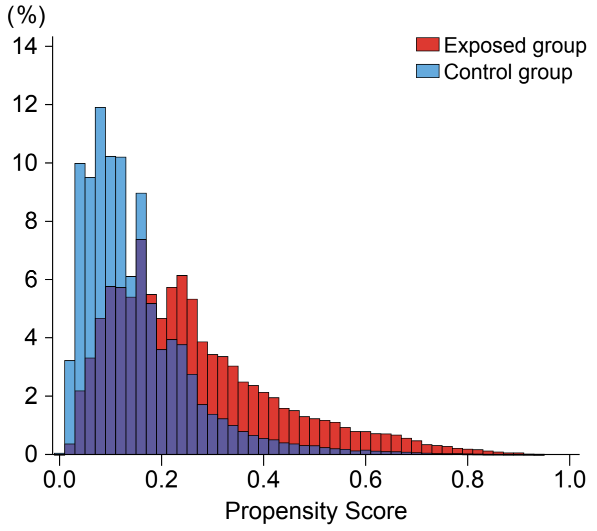

J After applying SMRW method:
PP analysis (gap or grace period for PP analysis is 180 days)

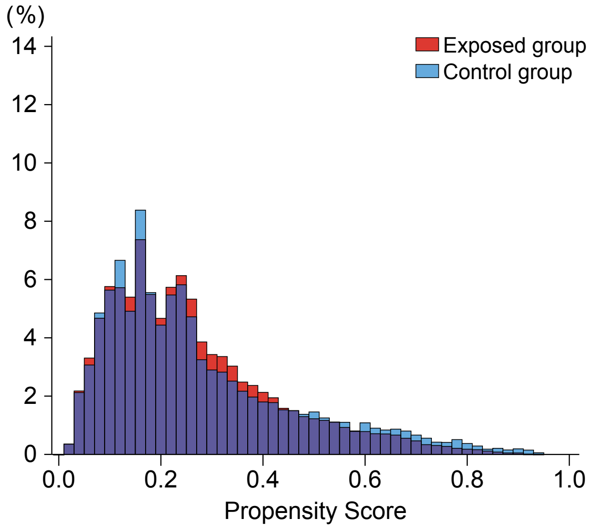

ITT, intention-to-treat; PP, per-protocol; SMRW, standardized mortality ratio weight.

Supplemental Figure 3. Kaplan-Meier plot of time to first VTE event

A PP analysis (gap or grace period for PP analysis is 0 days)

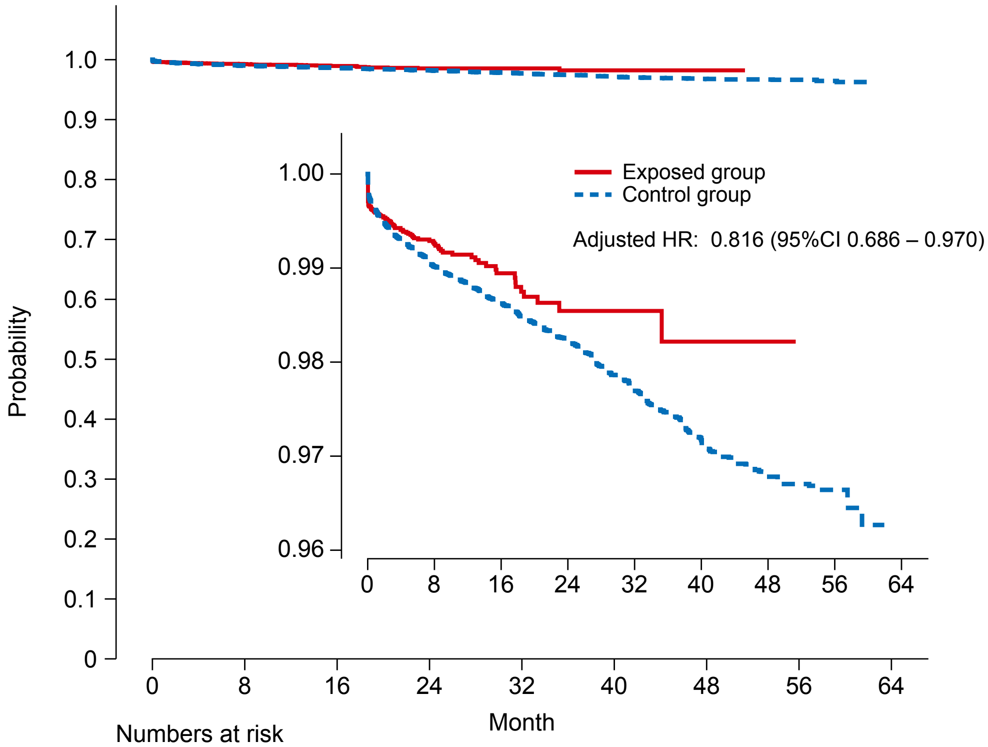

Numbers at risk

	0	8	16	24	32	40	48	56
Exposed group	23,195	5966	2435	1016	468	171	10	
Control group	23,584	15,327	10,687	7158	4513	2553	1127	224

B PP analysis (gap or grace period for PP analysis is 30 days)

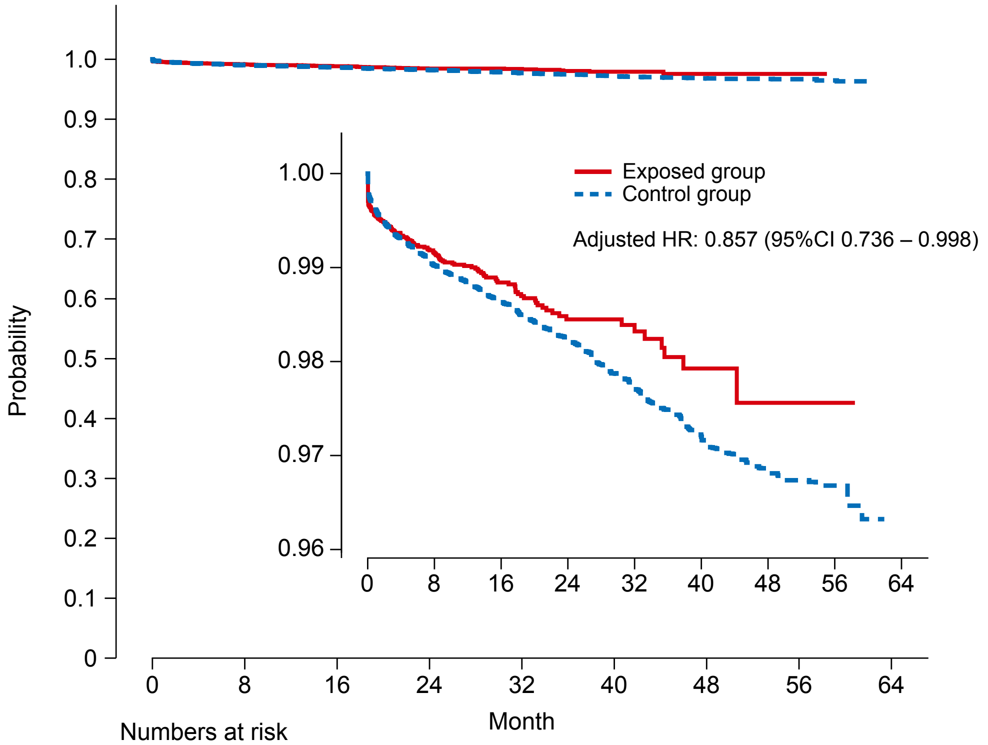

Numbers at risk

	0	8	16	24	32	40	48	56
Exposed group	23,195	9601	5347	2816	1433	615	51	3
Control group	23,586	15,347	10,698	7161	4512	2555	1126	226

Supplemental Figure 3. Continued.

C PP analysis (gap or grace period for PP analysis is 90 days)

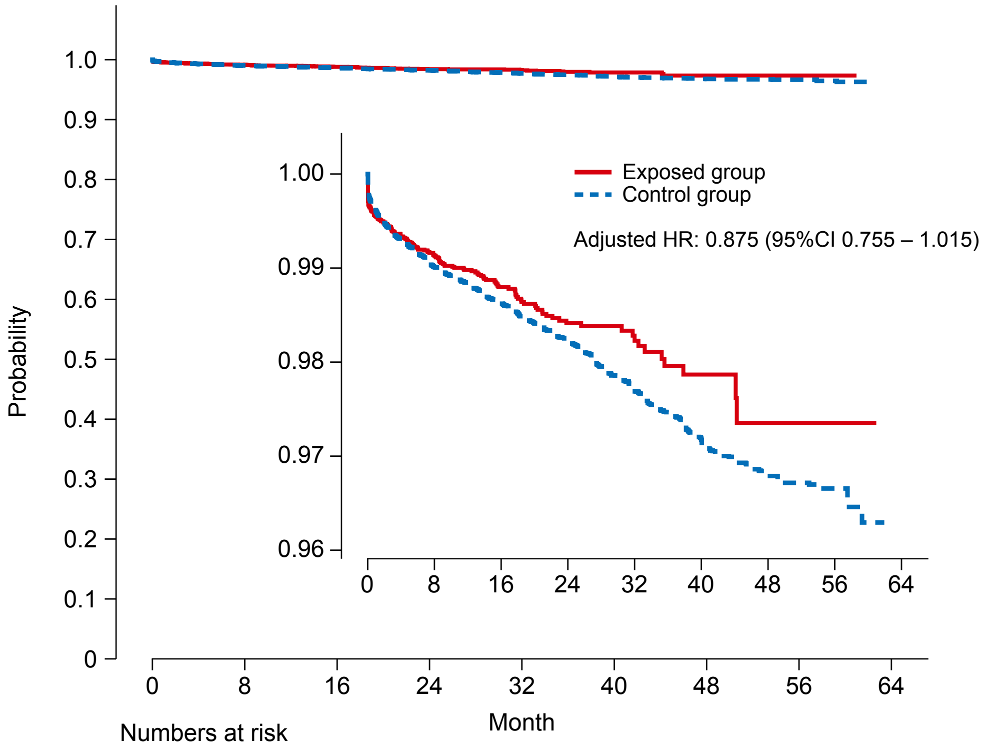

Numbers at risk								
Exposed group	23,195	10,539	6288	3499	1825	834	80	6
Control group	23,587	15,344	10,698	7170	4529	2568	1135	228

D PP analysis (gap or grace period for PP analysis is 180 days)

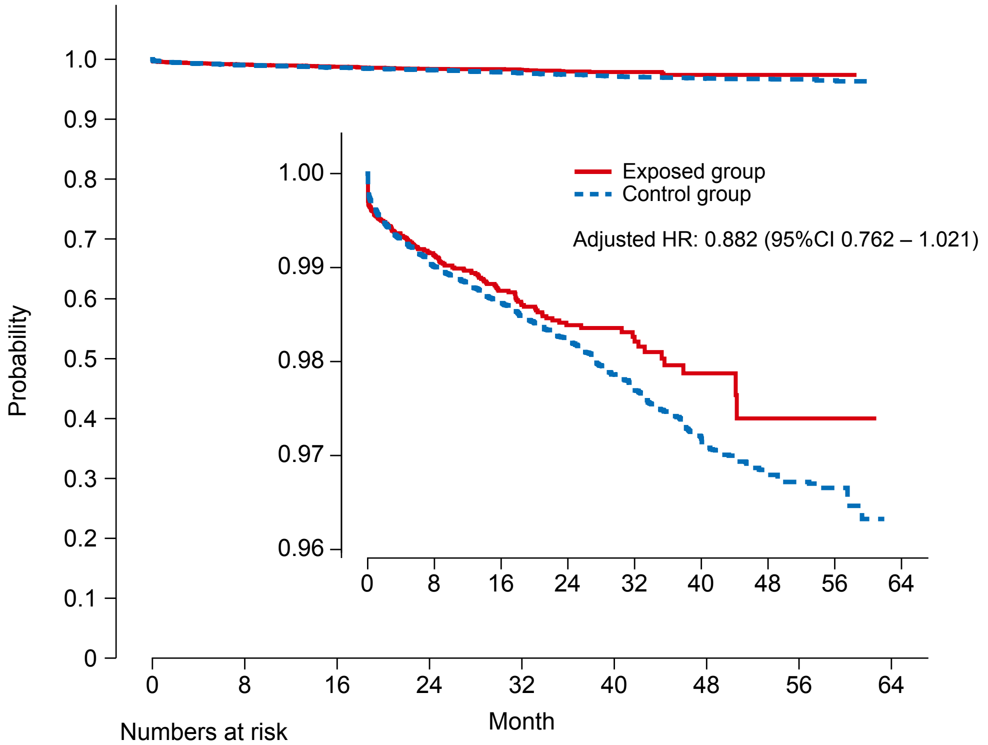

Numbers at risk								
Exposed group	23,195	10,895	6546	3675	1938	899	86	7
Control group	23,586	15,346	10,699	7164	4525	2569	1134	228

Cox proportional hazards model weighted with SMRW.

CI, confidence interval; HR, hazard ratio; PP, per-protocol; SMRW, standardized mortality ratio weight; VTE, venous thromboembolism.

Supplemental Figure 4. Forest plot of time to first VTE event for subgroup: ITT analysis

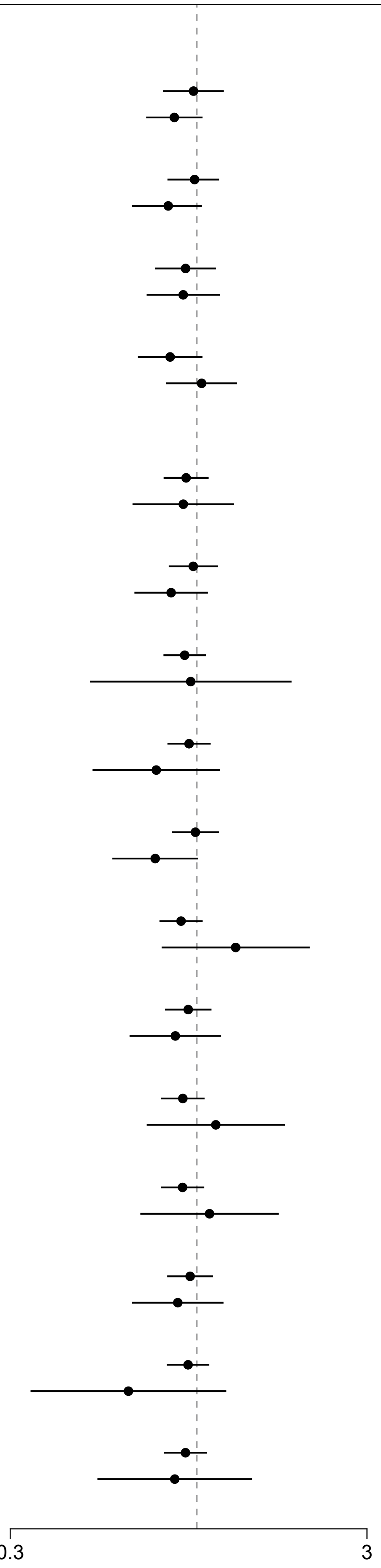

Subgroup	N	Adjusted HR (95%CI)
Demographics		
Age		
<Median	67,522	0.979 (0.806 – 1.191)
≥Median	71,618	0.865 (0.721 – 1.038)
Sex		
Male	74,362	0.978 (0.828 – 1.155)
Female	64,808	0.825 (0.658 – 1.033)
BMI		
<25	86,227	0.930 (0.764 – 1.132)
≥25	52,943	0.916 (0.724 – 1.160)
Smoking history		
No	88,506	0.842 (0.684 – 1.037)
Yes	50,664	1.032 (0.821 – 1.298)
Medical history		
Hypertension		
No	119,773	0.934 (0.807 – 1.080)
Yes	19,397	0.917 (0.661 – 1.272)
Diabetes mellitus		
No	103,736	0.977 (0.834 – 1.145)
Yes	35,434	0.848 (0.669 – 1.075)
Chronic kidney disease		
No	134,951	0.925 (0.807 – 1.061)
Yes	4219	0.962 (0.502 – 1.844)
Liver disease		
No	127,654	0.952 (0.828 – 1.094)
Yes	11,516	0.770 (0.510 – 1.163)
Cancer		
No	111,821	0.991 (0.852 – 1.154)
Yes	27,349	0.765 (0.580 – 1.009)
Myocardial infarction		
No	132,806	0.904 (0.786 – 1.039)
Yes	6364	1.286 (0.797 – 2.073)
Heart failure		
No	121,767	0.946 (0.814 – 1.100)
Yes	17,403	0.871 (0.648 – 1.170)
Atrial fibrillation/flutter		
No	135,243	0.914 (0.795 – 1.052)
Yes	3927	1.131 (0.724 – 1.766)
Cerebrovascular disease		
No	127,828	0.912 (0.793 – 1.050)
Yes	11,342	1.086 (0.695 – 1.698)
Pneumonia		
No	117,829	0.958 (0.826 – 1.111)
Yes	21,341	0.885 (0.659 – 1.188)
Chronic pulmonary disease		
No	134,176	0.945 (0.824 – 1.084)
Yes	4994	0.643 (0.342 – 1.210)
Urinary tract infection		
No	131,512	0.930 (0.809 – 1.069)
Yes	7658	0.868 (0.527 – 1.429)

Supplemental Figure 4. Continued.

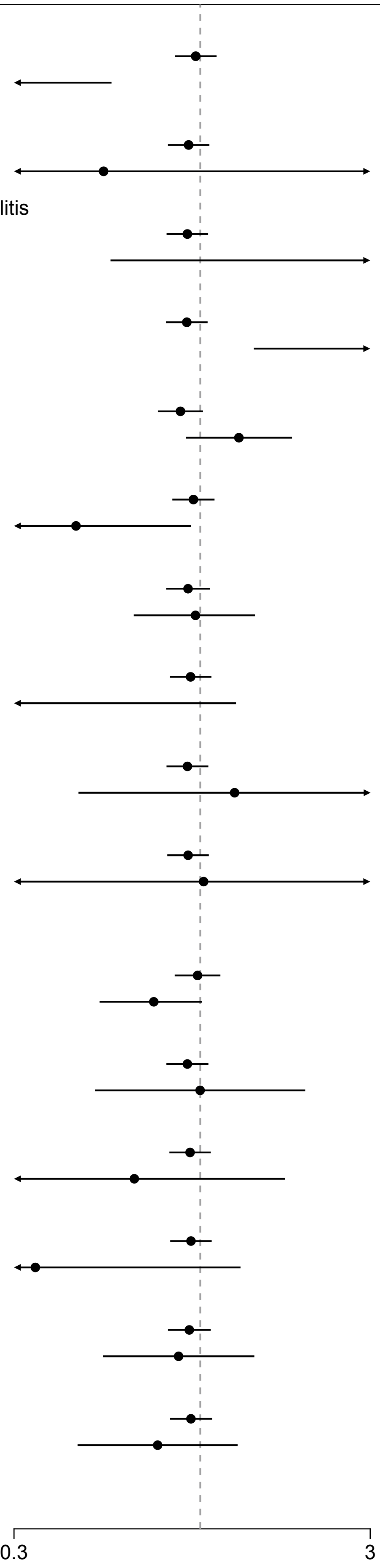

Subgroup	N	Adjusted HR (95%CI)
Connective tissue disease		
No	135,112	0.971 (0.849 – 1.111)
Yes	4058	0.173 (0.053 – 0.563)
HIV		
No	138,541	0.928 (0.811 – 1.061)
Yes	629	0.535 (0.071 – 4.034)
Crohn's disease or ulcerative colitis		
No	139,022	0.920 (0.805 – 1.052)
Yes	148	3.490 (0.560 – 21.757)
Varicose Vein		
No	138,928	0.917 (0.801 – 1.049)
Yes	242	5.859 (1.414 – 24.272)
Coagulopathy		
No	129,815	0.880 (0.761 – 1.018)
Yes	9355	1.284 (0.911 – 1.809)
Fracture, trauma, injury		
No	132,780	0.957 (0.835 – 1.097)
Yes	6390	0.448 (0.213 – 0.943)
Surgery		
No	125,851	0.924 (0.802 – 1.065)
Yes	13,319	0.963 (0.651 – 1.425)
Blood transfusion		
No	137,383	0.939 (0.821 – 1.075)
Yes	1787	0.291 (0.067 – 1.260)
Central venous line		
No	138,061	0.920 (0.804 – 1.054)
Yes	1109	1.246 (0.454 – 3.417)
Intravenous catheter or lead		
No	138,983	0.924 (0.808 – 1.057)
Yes	187	1.022 (0.119 – 8.751)
Concomitant use		
Statin		
No	104,439	0.983 (0.848 – 1.139)
Yes	34,731	0.732 (0.536 – 0.999)
Ezetimibe		
No	134,541	0.920 (0.803 – 1.054)
Yes	4629	0.999 (0.507 – 1.971)
IPE		
No	136,821	0.936 (0.819 – 1.070)
Yes	2349	0.653 (0.246 – 1.731)
ω-3 fatty acid		
No	137,953	0.942 (0.824 – 1.077)
Yes	1217	0.344 (0.091 – 1.297)
Insulin		
No	132,047	0.932 (0.812 – 1.069)
Yes	7123	0.869 (0.533 – 1.419)
Metformin		
No	129,977	0.942 (0.821 – 1.079)
Yes	9193	0.759 (0.453 – 1.273)

Supplemental Figure 4. Continued.

Subgroup	N	Adjusted HR (95%CI)
Sulfonylurea		
No	136,553	0.931 (0.813 – 1.066)
Yes	2617	0.758 (0.324 – 1.773)
α-glucosidase inhibitor		
No	137,069	0.917 (0.801 – 1.050)
Yes	2101	1.345 (0.549 – 3.297)
SGLT2 inhibitor		
No	130,617	0.931 (0.811 – 1.069)
Yes	8553	0.866 (0.538 – 1.394)
DPP4 inhibitor		
No	126,509	0.966 (0.840 – 1.110)
Yes	12,661	0.704 (0.467 – 1.061)
GLP-1 agonist		
No	137,038	0.925 (0.810 – 1.057)
Yes	2132	0.814 (0.237 – 2.802)
ACE inhibitor/ARB		
No	112,045	0.921 (0.790 – 1.074)
Yes	27,125	0.934 (0.715 – 1.220)
β blocker		
No	123,200	0.943 (0.812 – 1.096)
Yes	15,970	0.890 (0.670 – 1.181)
Calcium channel blocker		
No	113,261	0.955 (0.821 – 1.110)
Yes	25,909	0.841 (0.633 – 1.118)
Chemotherapy		
No	132,420	0.939 (0.816 – 1.080)
Yes	6750	0.786 (0.499 – 1.238)
Hormone replacement therapy		
No	138,289	0.921 (0.805 – 1.053)
Yes	881	2.441 (0.471 – 12.654)
Platelet aggregation inhibitor		
No	119,683	0.947 (0.822 – 1.093)
Yes	19,487	0.813 (0.560 – 1.179)
Thrombolytic		
No	139,062	0.925 (0.809 – 1.058)
Yes	108	0.001 (0.000 – 0.004)
Oral anticoagulant		
No	131,682	0.870 (0.745 – 1.015)
Yes	7488	1.102 (0.840 – 1.445)
Parenteral anticoagulation		
No	131,199	0.949 (0.825 – 1.092)
Yes	7971	0.783 (0.498 – 1.230)

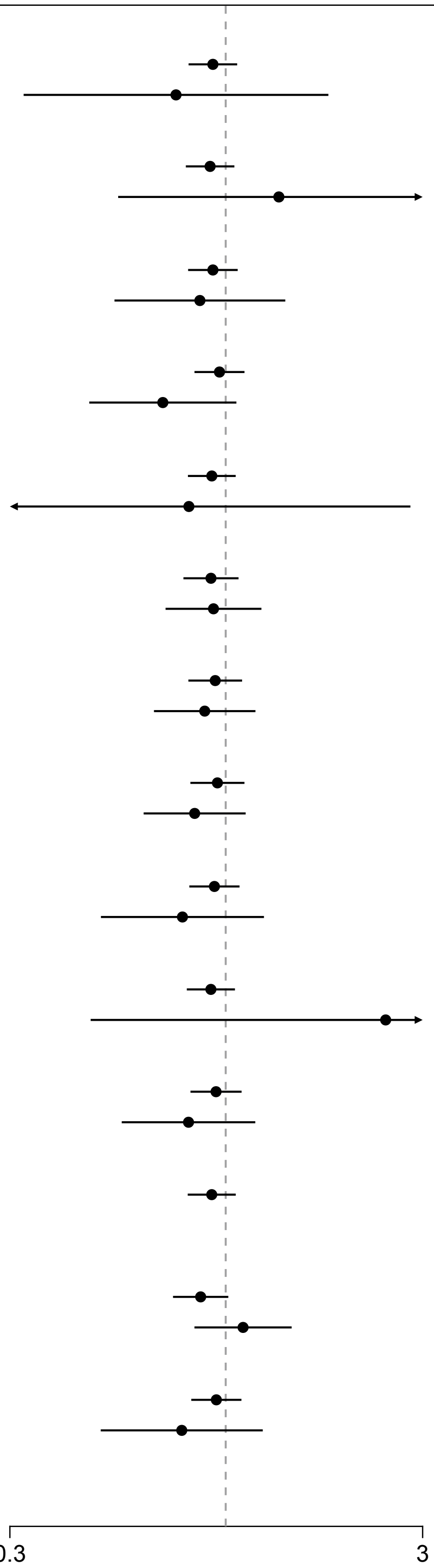

ACE, angiotensin converting enzyme; ARB, angiotensin II receptor blocker; BMI, body mass index; CI, confidence interval; DPP4, dipeptidyl peptidase-4; GLP-1, glucagon-like peptide-1; HIV, human immunodeficiency virus; HR, hazard ratio; IPE, icosapent ethyl; ITT, intention-to-treat; SGLT2, sodium glucose cotransporter 2; VTE, venous thromboembolism.
